# Global 5′-UTR RNA structure regulates translation of a *SERPINA1* mRNA

**DOI:** 10.1093/nar/gkac739

**Published:** 2022-09-15

**Authors:** Philip J Grayeski, Chase A Weidmann, Jayashree Kumar, Lela Lackey, Anthony M Mustoe, Steven Busan, Alain Laederach, Kevin M Weeks

**Affiliations:** Department of Chemistry, University of North Carolina, Chapel Hill, NC 27599-3290, USA; Department of Biological Chemistry, Center for RNA Biomedicine, University of Michigan Medical School, Ann Arbor, MI 48109, USA; Department of Biology, University of North Carolina, Chapel Hill, NC 27599, USA; Department of Genetics and Biochemistry, Center for Human Genetics, Clemson University, Greenwood, SC 29646, USA; Verna and Marrs McClean Department of Biochemistry and Molecular Biology, Department of Molecular and Human Genetics, and Therapeutic Innovation Center (THINC), Baylor College of Medicine, Houston, TX 77030, USA; Department of Chemistry, University of North Carolina, Chapel Hill, NC 27599-3290, USA; Department of Biology, University of North Carolina, Chapel Hill, NC 27599, USA; Department of Chemistry, University of North Carolina, Chapel Hill, NC 27599-3290, USA

## Abstract

*SERPINA1* mRNAs encode the protease inhibitor α-1-antitrypsin and are regulated through post-transcriptional mechanisms. α-1-antitrypsin deficiency leads to chronic obstructive pulmonary disease (COPD) and liver cirrhosis, and specific variants in the 5′-untranslated region (5′-UTR) are associated with COPD. The NM_000295.4 transcript is well expressed and translated in lung and blood and features an extended 5′-UTR that does not contain a competing upstream open reading frame (uORF). We show that the 5′-UTR of NM_000295.4 folds into a well-defined multi-helix structural domain. We systematically destabilized mRNA structure across the NM_000295.4 5′-UTR, and measured changes in (SHAPE quantified) RNA structure and cap-dependent translation relative to a native-sequence reporter. Surprisingly, despite destabilizing local RNA structure, most mutations either had no effect on or decreased translation. Most structure-destabilizing mutations retained native, global 5′-UTR structure. However, those mutations that disrupted the helix that anchors the 5′-UTR domain yielded three groups of non-native structures. Two of these non-native structure groups refolded to create a stable helix near the translation initiation site that decreases translation. Thus, in contrast to the conventional model that RNA structure in 5′-UTRs primarily inhibits translation, complex folding of the NM_000295.4 5′-UTR creates a translation-optimized message by promoting accessibility at the translation initiation site.

## INTRODUCTION

The *SERPINA1* gene encodes the protease inhibitor α-1-antitrypsin (A1AT) (1,2). A1AT is primarily expressed in the liver (3,4) and secreted into the vasculature where it circulates to the lung, and is expressed from the lung itself (4,5). The protein then functions to neutralize the activity of lung proteases and maintain lung plasticity (1,6). Post-transcriptional regulation of *SERPINA1* expression is complex (4). There are 11 known *SERPINA1* messenger RNA (mRNA) isoforms, generated via alternative splicing events that exclusively involve the 5′-untranslated region (5′-UTR) of the pre-mRNA (4,5). The 11 *SERPINA1* transcripts thus each contain a distinct 5′-UTR but encode the same protein sequence (4,5). RNA structure in the 5′-UTR has been shown to tune translation efficiency of individual *SERPINA1* mRNA isoforms by regulating ribosome accessibility to the start codons of primary and upstream open reading frames (uORFs) (4,7). Stable RNA structures reduce ribosome recognition and translation initiation at a start codon when located within roughly 15 nucleotides in either the 5′ or 3′ direction (4,7). In principle, the distinctive 5′-UTRs encoded by each *SERPINA1* transcript variant have the potential to encode translation start sites with distinct, individual accessibilities and translation efficiencies.

Dysregulation of *SERPINA1* is associated with chronic obstructive pulmonary disease, asthma and liver disease (8–11). The most well-studied dysregulation is A1AT deficiency, whereby missense mutations Glu342Lys and Glu264Val in A1AT account for approximately 96% of patients diagnosed with A1AT deficiency (12,13). Both mutations cause a toxic, concentration-dependent polymerization of misfolded protein in the liver, leading to cirrhosis, and insufficient secreted protease in the lung, leading to emphysema (8,14). Small decreases in *SERPINA1* expression in the liver, or small increases in the lung, are impactful in specific organ contexts (15,16). Additionally, large-scale clinical studies have shown that variability in patient A1AT serum levels is linked to mutations in *SERPINA1* 5′-UTR (non-coding) regions that alter translation, potentially through changes in RNA structure (17). Analyzing the extent to which these RNA-based mechanisms alter or restore physiological A1AT expression in the lung would inform future strategies to treat A1AT deficiency.

The NM_000295.4 *SERPINA1* isoform is one of the most expressed variants in the lung, accounting for 23% of total *SERPINA1* mRNA (Supplementary Figure S1). NM_000295.4 is also significantly expressed in primary tissue from spleen, blood, small intestine, kidney; and is one of the longest isoforms that does not contain an upstream open reading frame (uORF) (Supplementary Figure S1). uORFs generally compete with and reduce translation from the primary ORF, and therefore, the NM_000295.4 isoform is also likely to be among the most efficiently translated SERPINA1 mRNAs (4). NM_000295.4 is thus an important model system for understanding features that govern post-transcriptional gene regulation generally and for specifically dissecting the role of RNA structure in controlling translation.

**Figure 1.**
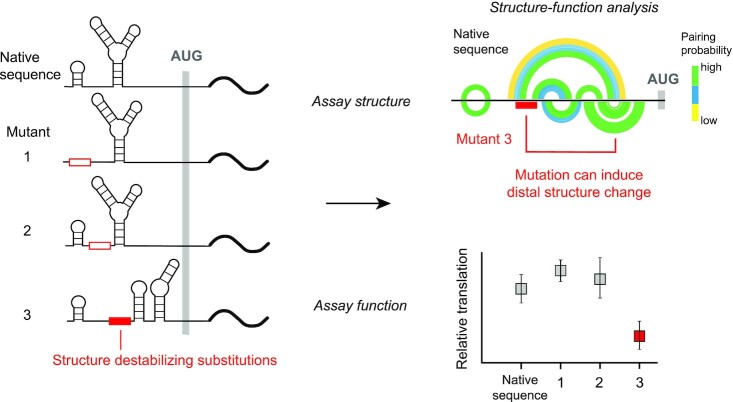
Strategy for analysis of structure-function relationships across a 5′-UTR. Structure-destabilizing substitutions (5′-UUAUUA-3′) were tiled across the SERPINA1 NM_000295.4 5′-UTR region in a luciferase reporter mRNA. Structural effects of mutation were assessed by nucleotide-resolution chemical probing (SHAPE-MaP) and data-directed structural modeling. Functional effects were evaluated in reporter translation assays.

Here, we implement a comprehensive strategy to examine RNA structure-function interrelationships by introducing consecutive six-nucleotide substitutions across the NM_000295.4 5′-UTR and then measuring both the structure and translation of each mutant RNA (Figure 1). RNA structures were examined using a SHAPE-based chemical probing strategy that enables accurate modeling of long and complex RNAs (18–21). We discovered that the 5′-UTR of NM_000295.4 is highly structured and only a minority of the introduced mutations altered the global architecture of the 5′-UTR. A subset of mutants, however, induced significant RNA refolding and reduced translation. Our work suggests that NM_000295.4 5′-UTR structure plays a productive role in translation, and preserves access to the translation initiation site to optimize cap-dependent translation. Our study provides a framework to explore the functional effects of large-scale 5′-UTR structure in other therapeutically relevant genes.

## MATERIALS AND METHODS

### Analysis of NM_000295.4 expression levels across human tissues

Relative NM_000295.4 expression levels were analyzed based on paired-end RNA-seq reads (22) from 6 different tissues (lung, spleen, blood, small intestine, kidney and liver; retrieved from the Genotype-Tissue Expression Project; dbGaP accession number phs000424.v8.p2). STAR was used to align reads to the human genome (23) and read depths at each nucleotide of the 5′-UTR were quantified using samtools (24). Read depth at every position was averaged across the total number of samples for each tissue. Average read depth across 50-nucleotides (chr14: 94 388 665–94 388 715) exclusive to the 5′-UTR exon of NM_000295.4 was calculated relative to the first 50-nucleotides (chr14: 94 383 191–94 383 241) of the first CDS exon to determine the abundance of NM_000295.4 relative to all other transcripts in each tissue (Supplementary Figure S1).

### Analysis of NM_000295.4 for non-canonical uORF translation

Potential non-canonical (non-AUG) initiation sites were analyzed using the NCBI *ORFfinder* (25). The native sequence 5′-UTR and each mutant 5′-UTR were examined for ORFs of 30 nucleotides or greater; all ORFs containing potential, efficient alternative initiation codons, particularly CUG and CUG/G initiation sequences (26,27), were analyzed. Three potential non-canonical uORF translation initiation sites and one termination site, 5′ of the start codon, are present in the native *NM_000295.4* sequence. Mutants overlapping these non-canonical translation initiation and termination sites were analyzed for translation impact. Mutating functional uORF initiation and functional termination sites is expected to increase and decrease translation, respectively. Mutations at these locations were observed to have no effect or counterintuitive effects. Specifically, several mutants—36, 108 and 180—disrupted potential, non-canonical uORF initiation sites, yet counterintuitively decreased translation by 14%, 17% and 31%. Mutant 144 disrupted the only potential non-canonical uORF termination site impacted by our mutational strategy and had no effect on translation. We conclude that none of the 5′-UUAUUA-3′ mutations introduced a distinct uORF initiation (including non-canonical sites) or altered termination of a non-canonical uORF in the native sequence.

### Plasmid construction

The plasmid backbone was pNL 3.2.CMV (Promega). Inverse PCR and religation was used to remove the PEST sequence following the nanoluciferase coding region (pPEST F1 and pPEST F2, primers; Supplementary Table S6). The protein produced from the resulting non-PEST nanoluciferase reporter has a half-life of 4 days in cell culture (28,29), similar to the 4–5 days half-life of A1AT (1,2). Thus, our nanoluciferase reporter without a PEST sequence is expected to model how global changes in RNA structure affect translation on timescales similar to that of the native A1AT protein. We replaced the nanoluciferase 5′-UTR with the native sequence NM_000295.4 5′-UTR and the first 240 nucleotides of the CDS and with mutant sequences. We then used inverse PCR and religation to substitute thymine for cytosine at the fifth nucleotide of the 5′-UTR for all mutants (pInv uORF F1 and pInv uORF R2M; Supplementary Table S6) to remove the previously described (non-functional) uORF of the NM_000295.4 isoform (4) (converting an ATG to ACG). This AUG is considered to be too close to the 5′-end of NM_000295.4 to function as a uORF in the native transcript (4); however, the reporter construct used here adds CMV promoter sequences (45 nts) to the 5′-end that could make this uORF functional in translation assays. We also removed the translation start site of the nanoluciferase gene using inverse PCR and religation (pInv ATG F1 and pInv ATG R2; Supplementary Table S6). Sequences were confirmed with full-plasmid sequencing. Production of the desired full-length transcripts, from the 5′-UTR to the poly-A tail, were confirmed by Sanger sequencing for all mutants (Dataset S1). Plasmid replicates were isolated from independent bacteria colonies, transfected with sequence-confirmed plasmid.

### Cell culture

HEK293T and HepG2 cells were cultured in minimum essential media (MEM, ThermoFischer) with 10% fetal bovine serum (FBS). For chemical probing experiments, HEK293T and HepG2 cells were treated at 70–80% and 30–50% confluence, respectively. HepG2 cells were grown and treated at a lower density to reduce spheroid formation and promote 5-nitroisatoic anhydride (5NIA) permeability (30). For biological replicates, experiments were performed on distinct populations of cells on different days.

### Transfection of NM_000295.4 variant plasmids for multiplexed chemical probing experiments

HEK293T cells were plated at 100 000 cells per well in six-well plates in 3 ml of growth media. Plated cells were cultured for 24 h at 37°C. Cells were then transfected with one of three pools of plasmids encoding mutant 5′-UTRs. Each pool was a 113-ng mixture of reporter plasmids, comprised of 9.4 ng each of the native sequence construct and 14 unique mutant constructs. Each well was transfected with 3 μg of plasmid [113 ng of reporter plasmids, 2888 ng of carrier (E488B, Promega)] in 10 μl of transfection reagent (Fugene 6, Promega). Cells were cultured at 37°C for 24 h before chemical probing experiments.

### In-cell treatment with 5NIA SHAPE reagent

HEK293T and HepG2 cells were grown in six-well plates. In-cell 5NIA treatment was performed as described (30,31). Cells were washed once in PBS, and then covered with 900 μl of serum-free MEM. We then added 100 μl of 250 mM 5NIA (Astatech) in anhydrous DMSO and gently mixed. To no-reaction controls, we added 100 μl of neat DMSO. Cells were treated with 5NIA (or neat DMSO) for 10 min at 37°C. Cells were then washed once with 1 ml of PBS. RNA was harvested using TRIzol (Invitrogen).

### Cell-free treatment with 5NIA SHAPE reagent

HEK293T and HepG2 cells were grown in six-well plates and washed once in PBS. The following procedure is designed to deproteinize RNA while avoiding harsh chemical denaturants to maintain native-like RNA structure (32). SHAPE treatment of gently extracted RNA was performed as described (20,31,32). Briefly, cells were resuspended in 750 μl of lysis buffer [40 mM Tris–HCl (pH 8.0), 25 mM NaCl, 6 mM MgCl_2_, 1 mM CaCl_2_, 256 mM sucrose, 0.5% Triton X-100, 1000 units/ml RNasin (Promega), 450 units/ml DNase I (Roche)]. Cells were lysed for 45 min at 23°C with agitation in cell plates. RNA was extracted twice with one volume of phenol:chloroform:isoamyl alcohol (25:24:1, v/v, Thermofisher) that had been pre-equilibrated with 1.1× folding buffer (111 mM HEPES (pH 8.0), 111 mM NaCl, 5.55 mM MgCl_2_), followed by two extractions with one volume of chloroform. RNA was buffer exchanged into 1.1× folding buffer over a desalting column (PD-10, GE Healthcare). RNA was then incubated at 37°C for 20 min and split into two equal portions. One portion was added to a 1/9 volume of 250 mM 5NIA in DMSO, and the other was added to a 1/9 volume of neat DMSO. Both portions were incubated for 10 min at 37°C.

### RNA precipitation and DNase treatment

Nucleic acids, from both in-cell and cell-free chemical probing experiments, were precipitated by addition of 1 volume of isopropanol and 1/20 volume of 4 M NaCl for 10 min at 23°C. Centrifugation at 10 000 × g at 4°C for 10 min formed an RNA pellet. Precipitates were washed once in 75% ethanol and pelleted again by centrifugation at 7500 × g at 4°C for 5 min. Pellets were resuspended in 100 μl of 1× DNase buffer and incubated with 1 unit of DNase (TURBO, Thermo Fisher) at 37°C for 30 min. After the first incubation, 1 more unit of DNase was added, and samples were incubated at 37°C for an additional 30 min. The RNA was then recovered by affinity bead purification (Mag-Bind TotalPure NGS SPRI beads, Omega Bio-tek; 1.8× volume of bead solution:DNase reaction).

### MaP reverse transcription

MaP reverse transcription was performed as described (20,33). For both endogenous and plasmid NM_000295, 2 pmol of gene-specific primer (1 μl of 2 μM of primer) was mixed with 1 μg of total RNA for an 8 μl RNA-primer mix (Supplementary Table S6). To RNA-primer mixes, 2 μl of 10 nM dNTPs were added and heated to 68°C for 5 min, and then immediately placed at 4°C for 2 min. To this template solution, 9 μl of freshly-made 2.22× MaP buffer [111 mM Tris (pH 8.0), 167 mM KCl, 22 mM DTT, 6 mM MnCl_2_, 2.22 M betaine] was added, and the mixture was incubated at 23°C for 2 min. SuperScript II reverse transcriptase (200 units, Thermo Fisher) was added, and reaction mixtures were incubated at 25°C for 10 min, 42°C for 90 min, 10 × [50°C for 2 min, 42°C for 2 min], and 72°C for 10 min to inactivate enzyme. Reverse transcription reactions were buffer exchanged into TE buffer [10 mM Tris–HCl (pH 8.0), 1 mM EDTA] using G-50 microspin columns (Illustra, GE Healthcare).

### Two-step PCR of small RNA MaP libraries

Sequencing libraries were prepared from cDNA libraries using a two-step PCR strategy (34). For endogenous-specific amplicons, 3 μl of cDNA template was amplified in Step 1 PCR using a 25-cycle gene-specific PCR temperature program: 98°C for 30 s, 20 × [98°C for 5 s, 65°C for 30 s, 72°C for 20 s], 72°C for 2 min (Supplementary Table S6). A 5′ primer for the endogenous gene was selected that optimized amplification of the endogenous gene. For plasmid-specific amplicons, 3 μl of cDNA template was amplified in 20 cycles of the same temperature program. The plasmid amplicon has an additional CMV-element that allowed for a 5′-primer to efficiently bind and amplify of the entire native sequence. Step 1 PCR products were purified (SPRI beads, Mag-Bind TotalPure NGS, Omega Bio-tek, at a 0.8× ratio) and eluted in water. In the second PCR step, 2 ng of Step 1 PCR product was amplified with treatment-specific barcodes with the following temperature program: 98°C for 30 s, 10 × [98°C for 5 s, 68°C for 20 s, 72°C for 20 s], 72°C for 2 min. Step 2 PCR products were purified (SPRI beads, Mag-Bind TotalPure NGS, Omega Bio-tek, at a 0.8× ratio) and eluted in water.

### Sequencing, mutation counting and SHAPE profile generation

Amplicon libraries were verified for correct size and purity (Agilent 2100 Bioanalyzer). Step 2 PCR products were sequenced with 2 × 300 paired-end sequencing (MiSeq, Illumina). For sequencing libraries from endogenous NM_000295.4 amplification, ShapeMapper (v.2.1.4) was used to align reads, calculate mutation rates from MaP, and generate SHAPE profiles with default parameters (35). For sequencing libraries from plasmid reporters, the ShapeMapper (v.2.1.4) alignment function was modified to perform paired-end alignment using *Bowtie2* with the following arguments: –local -D 15 -R 3 -N 1 -L 20 -i S,1,0.50. ShapeMapper calculated apparent mutation rates from MaP data to generate SHAPE reactivity profiles (35). Median read-depths for all SHAPE-MaP samples and controls was >20 000; nucleotides with a read depth <4000 were excluded from analysis.

### Calculating average per-nucleotide SHAPE reactivity across biological replicates

**Figure 2.**
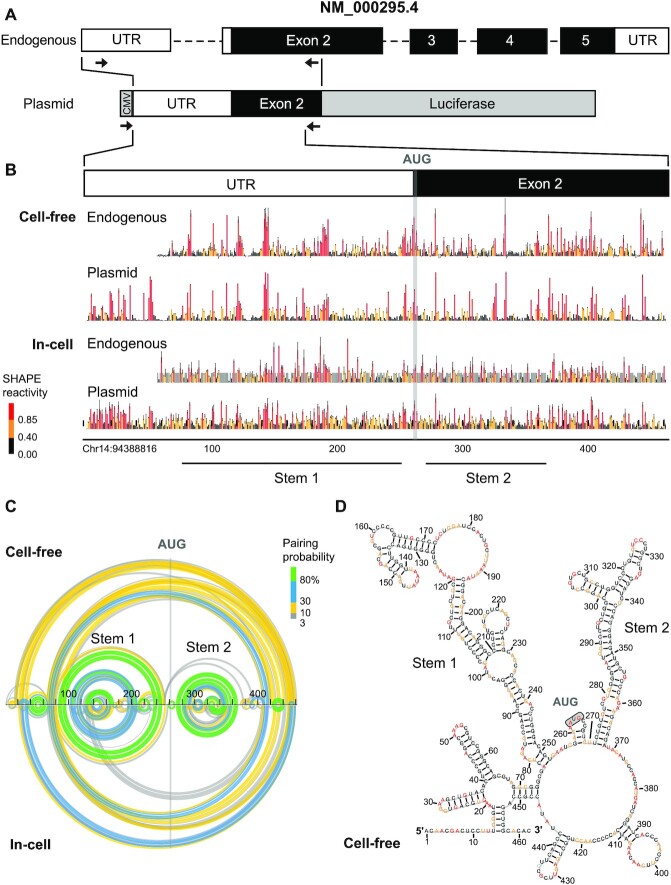
Structure of the NM_000295.4 5′-UTR and CDS. (**A**) Structures of the pre-mRNA of *SERPINA1* isoform NM_000295.4 and plasmid reporter. The reporter mRNA contains the entire spliced 5′-UTR and 240 nucleotides of the CDS, inserted downstream of a CMV promoter (45 nucleotides). Arrows indicate primers used to selectively analyze endogenous and plasmid-based mRNAs. (**B**) SHAPE profiles for the endogenous NM_000295.4 mRNA expressed in hepatocytes (HepG2), and for the native sequence reporter construct, expressed in HEK293T cells. Data for cell-free and in-cell probing are shown. The transcription start site for NM_000295.4 is annotated as +1. Short gray bars indicate nucleotides with high inter-replicate variability (>50%) for in-cell experiment with endogenous RNA. (**C**) Arc diagrams showing pairing probabilities for base pairs modeled under cell-free and in-cell conditions for the native sequence NM_000295.4 5′-UTR and CDS, encoded by the reporter plasmid. Pairing probabilities are indicated by color scale. (**D**) Secondary structure model for native sequence NM_000295.4 5′-UTR and CDS (240 nucleotides) under cell-free conditions. Nucleotides are colored by SHAPE reactivity. The overall structure is conserved between endogenous and plasmid-encoded RNAs under both cell-free and in-cell conditions (Supplementary Figure S2, Tables S1 and S2).

The arithmetic mean and standard error of the mean for the normalized SHAPE reactivity of each nucleotide were calculated across biological replicates to generate a nucleotide-averaged MaP file. These MaP files were visualized using arcPlot (https://github.com/Weeks-UNC/arcPlot). If the standard error was ≥50% of the arithmetic mean for a specific nucleotide, nucleotides were masked to indicate high inter-replicate variability. This high inter-replicate variability was observed only for a subset of nucleotides in the in-cell treatment of HepG2 cells for the endogenous gene (gray bars, in Figure 2 and Supplementary Figure S2).

### Secondary structure modeling

RNA structure modeling was performed using Superfold (32), which uses SHAPE reactivity data to inform RNAStructure (v5.8) modeling (36). Default parameters were used to generate pairing probabilities and minimum free energy structures as follows: SHAPEslope = 1.8, SHAPEintercept = −0.6, trimInterior = 300, partitionWindowSize = 1200, partitionStepSize = 100, foldWindowSize = 3000, foldStepSize = 300, maxPairingDist = 600. Secondary structure diagrams were generated using the visualization applet for RNA (VARNA) (37).

### Dual-luciferase assay for relative translation of NM_000295 5′-UTR mutants

HEK293T cells were plated at 10,000 cells per well in nine six-well plates in 100 μl of growth media and then cultured for 24 h at 37°C. Each well was then transfected with a mixture of 80 ng of plasmid (42 ng of carrier plasmid (E488B, Promega), 35 ng firefly plasmid, and 3 ng of reporter plasmid), and 0.24 μl of transfection reagent (FuGENE 6, Promega). After culture for 21 h, 50 μl of media was aspirated from each well, and 50 μl of firefly substrate solution (ONE-Glo Ex Reagent, Promega) was added to each well. Samples were incubated for 30 min at 23°C. A 50-μl aliquot of the nanoluciferase substrate solution (NanoDLR Stop & Glo Reagent, Promega) was then added to each well. Samples were incubated for another 30 min at 23°C. Firefly and nanoluciferase luminescence were measured using standard luminescence protocols (Clariostar Microplate Reader, BMG Labtech).

### Calculation of direct reactivity

Measures of direct reactivity (Supplementary Table S1) were computed as described (38). Direct reactivity was calculated as ln(rateMod/rateUnt), where rateMod and rateUnt refer to MaP rates in 5NIA-treated (modified) and untreated controls, respectively. Nucleotides with read depths below 4000 were excluded.

### Calculation of structural model similarity

The computeSensPPV program of RNATools (v. 2.0) was used to calculate sensitivity (sens) and positive predictive value (ppv) of shared base pairs across RNA structure models (32,36). The arithmetic means of sens and ppv (with a pairing probability cutoff of 0.1) were used as measures of ‘overall similarity’ between RNA structure models.

### ΔSHAPE of native sequence and mutant cell-free 5′-UTR

Normalized SHAPE reactivities for each mutant sequence were compared to the native sequence using ΔSHAPE (39). Default parameters were used and 5′ and 3′ primer sequences were excluded from analysis. Differences were determined to be significant using *Z*-factor and standard scoring significance testing.

### Principal component analysis and clustering of mutant 5′-UTR structures

Overall similarity between RNA structure models were calculated (arithmetic mean of sens and ppv) as described (32,36). Only base pairs within the 5′-UTR, from nucleotide +1 to +270, were analyzed. Overall similarity values between all native sequence and 42 mutant RNA structure models were input into a 43 × 43 matrix. Principle components were generated from the 43 × 43 matrix, using the *pca* package of *sklearn* (https://scikit-learn.org/stable/). These structures were projected onto the first two principal components and visualized as a 2D scatterplot (40,41). Using the *cluster* package from *sklearn*, inertia was used to calculate the optimal *k* value for clustering the distribution, and a *k*-means clustering algorithm identified four distinct groups and the centroid structure of each group (40,41).

### Analysis of translation

Nanoluciferase (NL) was normalized to firefly (FF) to calculate a NL/FF ratio. This normalization controlled for transfection variability and cell viability. Mutant NL/FF values were calculated relative to the native sequence NL/FF value. The arithmetic average and standard deviation of relative mutant translation changes was calculated across three biological replicates for two plasmid replicates of each mutant (*n* = 6) (Dataset S2).

### Analysis of cap-dependence of NM_000295.4-nanoluciferase translation

The 5′-cap dependence for translation of the NM_000295.4 nanoluciferase reporter was examined through a luciferase reporter assay in the presence of a cap-dependent translation inhibitor, 4E1RCat (PubChem ID 16195554, Sigma Aldrich). 4E1RCat disrupts interactions of eukaryotic initiation factor 4E (the cap binding protein) with initiation factors 4G and 4E-BP1 at low micromolar potencies (42). HEK293T cells were plated at 10 000 cells per well in 96-well plates in 100 μl of growth media and then cultured for 24 h at 37°C. Each well was transfected with a mixture of 80 ng of total plasmid [42 ng of carrier plasmid (E488B, Promega), 35 ng firefly plasmid, and 3 ng of reporter plasmid] and 0.24 μl of transfection reagent (FuGENE 6, Promega). Firefly plasmid was included for consistency with dual-luciferase assays analyzing mutant translation. After culture for 17 h, 100 μM 4E1RCat in DMSO was added to the wells (42). Untreated controls contained an equivalent volume of DMSO. After culture for 21 h, 50 μl of media was aspirated from each well, and 50 μl of firefly substrate solution was added (ONE-Glo Ex Reagent, Promega). Samples were incubated for 30 min at 23°C. A 50-μl aliquot of the nanoluciferase substrate solution (NanoDLR Stop & Glo Reagent, Promega) was then added to each well. Samples were incubated for another 30 min at 23°C. Nanoluciferase luminescence was measured using standard luminescence protocols (Clariostar Microplate Reader, BMG Labtech). Translation of the nanoluciferase reporter was reduced by 85% and 86% in HEK293T cells in the presence of 100 μM 4E1RCat after 4 h across two biological replicates, respectively.

### *Calculation of* Δ*G*^‡^_unfold_

Non-equilibrium Δ*G*^‡^_unfold_ was calculated as described (4,19). This calculation measures the cost of disrupting a specific RNA structure and does not allow the RNA to refold. This non-equilibrium model provides the strongest correlation with translation efficiency (4,19). The free energy of a ‘constrained’ transcript, in which the translation initiation site is constrained to be single-stranded, is compared to the free energy of a reference transcript:

$$\begin{equation*}{\rm{\Delta }}{{{G}}^{\rm{\ddagger }}}_{{\rm{unfold}}}{\rm{ = \Delta }}{{{G}}_{{\rm{constrained}}}}{\rm{ - \Delta }}{{{G}}_{{\rm{reference}}}}\end{equation*}$$

The SHAPE-directed minimum free energy structure of each mutant was used as the reference. The constrained structure was generated by removing base pairs within ±13 to ±16 nucleotides from the adenosine of the start codon of the reference structure (Dataset S2). Values for ±15 nucleotides were consistent with prior analysis of *SERPINA1* translation (4). Calculations were performed using the *efn2* command from RNAStructure (v.5.8) on SHAPE-directed structure files (.ct files) (36).

## RESULTS

### The NM_000295.4 5′-UTR is highly structured

The *SERPINA1* locus has 11 annotated transcripts (Supplementary Figure S1A), of which the NM_000295.4 isoform features one of the longest 5′-UTRs without a canonical (AUG initiating) uORF (Supplementary Figure S1B) (5,17). NM_000295.4 shares a splicing pattern with NM_000295.5 but differs in its transcription start-site (Supplementary Figure S1B). Analysis of RNA-seq coverage maps for lung, spleen, blood, small intestine, kidney and liver tissues reveal substantial expression of both isoforms (based on all individuals in the Genotype-Tissue Expression database (43)). NM_000295.4 represents 23% of all lung-expressed SERPINA1 transcripts (Supplementary Figure S1C). Given the importance of A1AT to lung health and high expression of NM_000295.4 in lung tissue, we focused on structural characterization of the long-5′-UTR NM_000295.4 transcript and the impact of 5′-UTR structure on translation. Nonetheless, expression of *SERPINA1* 5′-UTR variants remains complex, with multiple transcription start sites and alternative splicing events (4).

We first examined the structure of the native, endogenous NM_000295.4 with SHAPE-MaP (20) using primers selective for the spliced NM_000295.4 transcript (Figure 2A). We probed the mRNA as gently extracted from cells (cell-free) (39,44) and in HepG2 cells (in-cell), derived from liver cells (30,39). HepG2 cells exhibit robust *SERPINA1* expression and are therefore a good model for the endogenous structure of the native 5′-UTR (45). Cell-free SHAPE data revealed that many nucleotides were unreactive, consistent with stable base pairing in both the 5′-UTR and CDS (Figure 2B). Broad features of the SHAPE reactivities were shared between the endogenous mRNA as examined under cell-free and in-cell conditions (Supplementary Table S1). Transcripts analyzed in cells had modestly higher SHAPE reactivities and greater experimental variability, consistent with effects of the in-cell environment such as transient unfolding by the ribosome during translation, and with challenges in working with HepG2 cells, which form aggregates in culture (Figure 2B).

We then created a plasmid-encoded native sequence reporter gene, fusing a nanoluciferase reporter gene sequence to the first 501 nucleotides of the mature, spliced NM_000295.4 isoform (Figure 2A). The inclusion of 240 nts of the *SERPINA1* CDS was designed to preserve native RNA folding and enables analysis of potential long-range structural interactions across the 5′-UTR and CDS. We measured cell-free and in-cell SHAPE reactivities for the plasmid-based native sequence reporter transcript in HEK293T cells (derived from kidney), which are more readily transfected than HepG2 cells. SHAPE data were highly reproducible and reactivity patterns for the endogenous cell-free and plasmid-based mRNAs were similar (Figure 2B) and showed good correlation (Supplementary Table S1). Thus, this model-free analysis of per-nucleotide SHAPE reactivities supports that our plasmid construct recapitulates the structural features of the endogenous mRNA.

SHAPE reactivity data can be used as pseudo-free energy terms to create data-directed RNA structural models (20,46). From our SHAPE data, we derived pairing probability profiles and minimum-free energy structures for the endogenous mRNA and for our plasmid-expressed native sequence NM_000295.4 mRNA. Cell-free and in-cell structure models shared large-scale features, defined by a core set of highly probable helices (Figure 2C, Supplementary Figure S2; Table S2). These helices included a short stem-loop near the 5′-end of the RNA (nucleotides 40–64) and two large stems, Stem 1 and Stem 2, each containing a three-helix junction. Stem 1 immediately precedes the Kozak sequence, and Stem 2 lies immediately after the translation start site (Figure 2C). Superimposing the SHAPE reactivities on the minimum free energy structure illustrates the complex experimentally-supported architecture of the NM_000295.4 5′-UTR (Figure 2D). The complexity of the 5′-UTR structure was surprising because NM_000295.4 is highly translated (4), and thermodynamically stable helices upstream of translation start sites are generally thought to repress translation (18,47,48).

### Exploring structure-function relationships in a *SERPINA1* 5′-UTR

We used a systematic mutational strategy to investigate structure-function relationships across the NM_000295.4 5′-UTR. All mutants were derived from our fused NM_000295.4-nanoluciferase reporter construct (Figure 2A). We introduced six-nucleotide substitutions of 5′-UUAUUA-3′ tiled across the NM_000295.4 5′-UTR upstream of the Kozak sequence (49), comprising a total of 42 mutant mRNAs. U/A substitutions were chosen to maximally disrupt base pairing in this G/C rich 5′-UTR (58% guanosine and cytidine nucleotides). We examined RNA structure and translation from transcripts produced from the native sequence reporter and the 42 mutants (Figure 1). This mutation strategy does not introduce uORFs or other known regulatory features (see Methods) and, as such, specifically and systematically interrogates the role of RNA structure in controlling translation.

This mutational strategy supported a multiplexed experimental and analytical approach. We measured mutation-induced structural changes in each 5′-UTR using SHAPE-MaP chemical probing experiments, where each mutation also functioned as a barcode enabling multiple RNAs to be probed together. Translation was assessed by measuring the luminescent output from individually transfected mutant reporter constructs in 96-well configuration. We were thus able to efficiently examine the relationship of RNA structure to translation in the 5′-UTR.

### Mutants form native-like and three alternative structure groups

**Figure 3.**
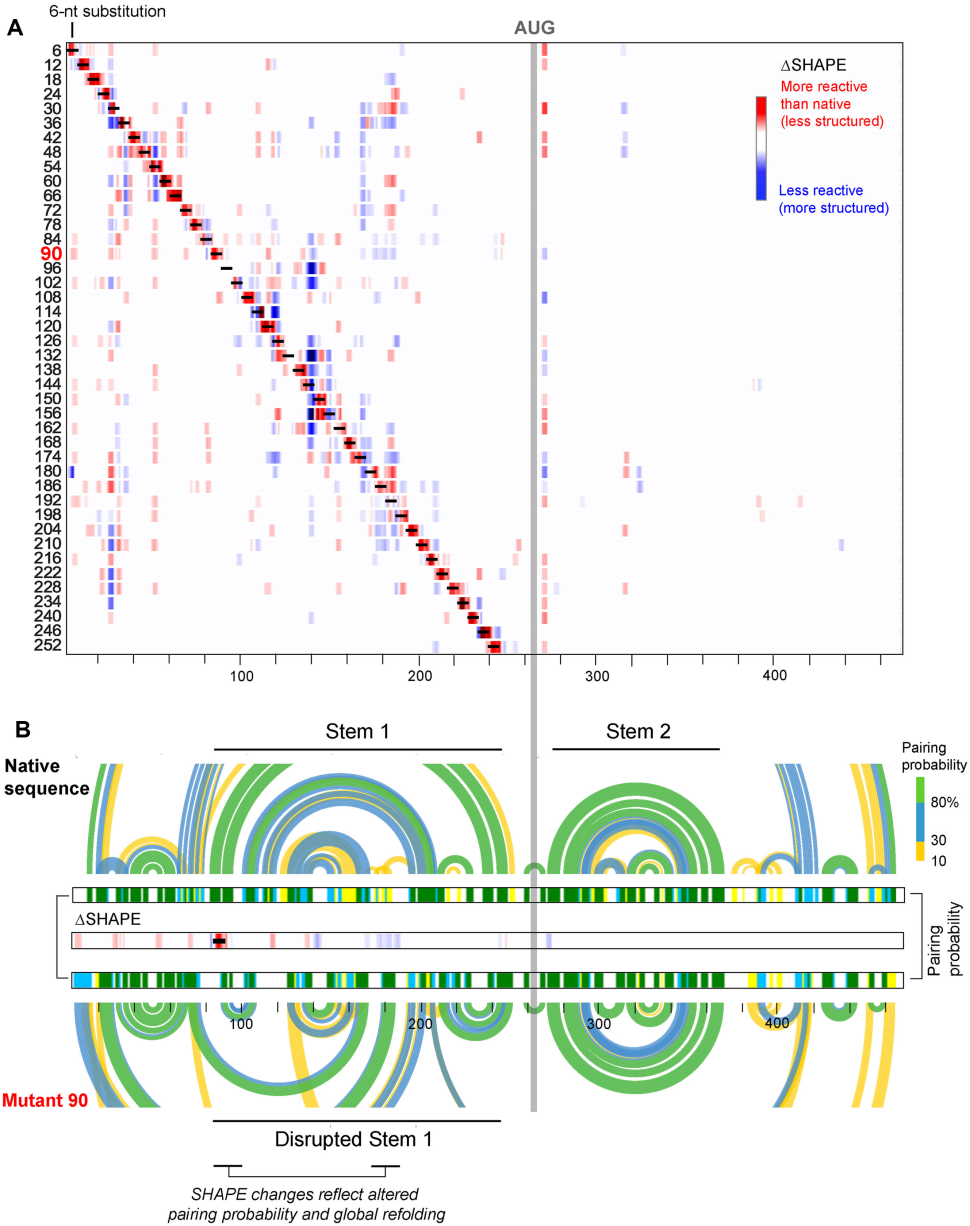
Consequences of local structure-destabilizing mutations across the NM_000295.4 5′-UTR and initial CDS. (**A**) Heat map of SHAPE reactivity changes—quantified as ΔSHAPE changes (44)—for each mutant relative to the native sequence transcript. Mutation sites are indicated by black bars; mutants are named by the position of their 3′-most substituted nucleotide. Increases and decreases are shown on a red to blue scale. (**B**) Arc diagrams, linear pairing probability plots, and ΔSHAPE (middle) for representative structure-altering mutant (mutant 90) versus native sequence transcript. Site of mutation indicated by black bar.

We obtained SHAPE data for all mutants under cell-free conditions. Experiments were performed in a multiplex format, transfecting 15 reporters (14 mutants and 1 native-sequence control) in three separate pools. SHAPE data were then deconvoluted using the mutated sequence as a barcode, which generated highly reproducible data across replicates (*R*^2^ > 0.9, Supplementary Table S3). We used the ΔSHAPE framework (44) to identify significant structural changes in each mutant relative to the native sequence. At each 5′-UUAUUA-3′ substitution site, we observed a local increase in SHAPE reactivity (Figure 3A, red differences), reflective of the elimination or weakening of local base pairs. Thus, the six-nucleotide substitution destabilized the immediate structure at each mutation site, as designed.

Most mutations caused only local changes in RNA structure, whereas others resulted in large-scale changes. For example, in mutants 6, 12, 18, 24, 54, 114, 138, 156, 216, 246 and 252 (names refer to the 3′-most position of the substitution mutation), significant ΔSHAPE changes were primarily limited to the site of the six-nucleotide substitution (Figure 3A). In contrast, in mutants 36, 42, 48, 90, 102, 198, 204, 210 and 228, we observed multiple changes in SHAPE reactivity in regions located up to 100 nucleotides or more from the mutation site. For example, mutant 90 (substitution at 85–90) showed local changes at the mutation site and decreases in SHAPE reactivity at nucleotides 175–186 (Figure 3A). Similarly, mutant 228 (substitution at 223–228) showed increased SHAPE reactivity at nucleotides 20–23 and 30–33, and a decrease at nucleotides 25–27 (Figure 3A).

Notably, large-scale structure changes occurred for a subset of mutants, but these changes were largely contained within the 5′-UTR. (Figure 3A). We do observe a few changes in ΔSHAPE signal immediately 3′ of the AUG signal, likely reflective of changes in the stability of the short stem-loop structure that spans the AUG region (Figure 3A). The clear overall lack of observed structure changes 3′ of the start codon is consistent with the SHAPE-directed structural model, which indicated that the major Stem 1 and Stem 2 RNA structures do not bridge the 5′-UTR and CDS (Figures 2D and 3B). Thus, the NM_000295.4 mRNA appears to fold into distinct 5′-UTR and CDS structural domains that behave independently.

Mutations that induced long-range or substantial local alterations in SHAPE reactivity relative to the native sequence construct also caused the RNA to fold differently. For example, pairing probabilities for mutant 90 differed considerably from those of the native sequence (Figure 3B, arc diagrams). Arc diagrams of pairing probabilities can be conveniently simplified and visualized as linear pairing probabilities. This visualization highlighted multiple changes in pairing probability across mutant 90 relative to the native sequence, as evidenced by a decrease between nucleotides 78–89 and 108–125 and an increase at positions 178–187 and 210–223 (Figure 3B, *center*, red and blue bands, respectively). Thus, the SHAPE reactivity changes in mutant 90 are consistent with local and distal changes in pairing probability, which support a substantially different overall RNA architecture.

SHAPE data were used to create structure models for all mutants. Structural models derived from chemical probing data were reproducible between replicates (Supplementary Table S3). Structural models derived without chemical probing data were often inconsistent with models derived from chemical probing data; in some cases, no-SHAPE models showed less than 15% agreement with SHAPE-informed structures, emphasizing the importance of (SHAPE) data for understanding the role of RNA structure in 5′-UTRs (Supplementary Table S4). Notably, many mutants formed distinct global structures (Supplementary Table S5).

**Figure 4.**
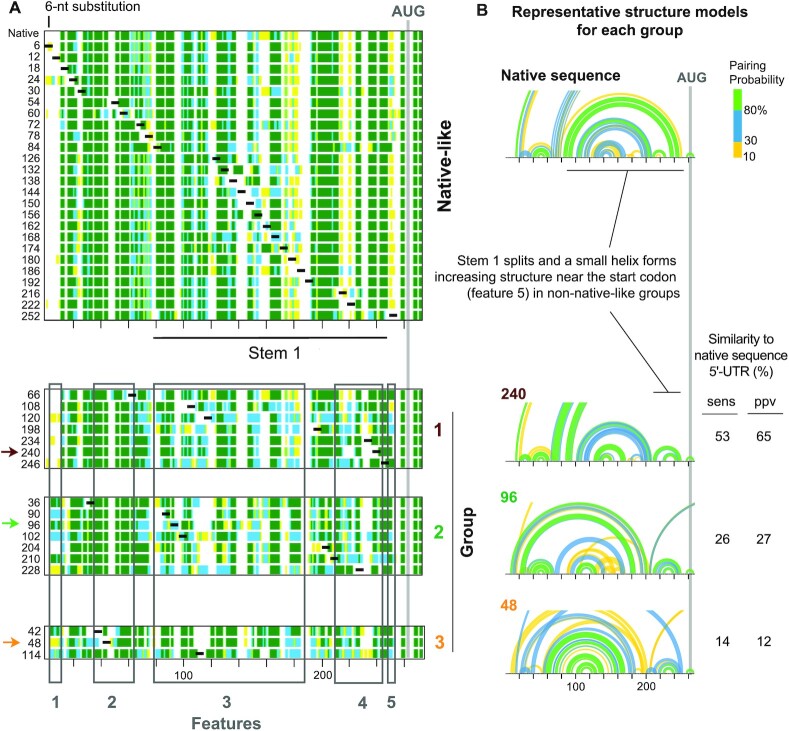
5′-UTR mutations form RNA structures that cluster into distinct groups. (**A**) Linear pairing probabilities for the native sequence and 42 mutant 5′-UTRs. A principal component analysis, based on similarity in pairing probabilities, yielded four clusters, native-like and groups 1, 2 and 3; groupings were supported by *k*-means clustering. Features, indicated by numbers below plots, denote regions with characteristic structural differences between groups. (**B**) Arc diagrams showing pairing probabilities for the native sequence structure and a representative mutant from each structure group. Sensitivity (sens) and positive predictive value (ppv) calculated from pairing probabilities (≥0.1 threshold) as compared to the native sequence 5′-UTR structure.

We used a principal component analysis to analyze similarity in pairing probabilities among the native sequence and 42 mutant 5′-UTRs. Analysis of the first two principal components revealed that the mutants fold into families of similar structures, defining four distinct groups based on *k*-means clustering (Figure 4A, Supplementary Figure S3). Structure models for 25 of the 42 mutants were similar to that of the native sequence (native-like group); however, other mutants (40%) had structural features that were distinct (groups 1–3; Figure 4).

Each of the three non-native groups have 5′-UTR structures that distinguished them from the native-like group (Figure 4B). Most noticeably, Stem 1 did not form in any of the non-native mutants, as revealed by the reduced pairing probability at nucleotides 75–185 (Figure 4A, feature 3; Figure 4B, arc diagrams). Group 2 and 3 mutants were more highly structured at the 5′-end of the RNA than the native-like 5′-UTRs (Figure 4A, feature 1). Group 3 mutants also lacked the small helix (nucleotides 40–64) preceding Stem 1 observed in the other groups (Figure 4A, feature 2). Further, all non-native mutants showed increased pairing probability at nucleotides 210–250, reflecting formation of a small helix that forms upon Stem 1 disruption (Figure 4A, features 4–5; Figure 4B, arc diagrams). Notably, this small helix in the non-native mutants increased structure near the start codon relative to the native-like group (Figure 4A, feature 5). In sum, 25 of the 42 mutants adopted structures similar to the native sequence NM_000295.4 5′-UTR; the non-native mutants all lacked Stem 1 and populated three discrete structural groups with distinct structural features.

### NM_000295.4 5′-UTR structure modulates translation

We first established that translation of the NM_000295.4 transcript occurs via a standard 5′-cap-dependent mechanism (48) and confirmed that our mutation strategy did not create potential non-canonical uORFs. We measured translation in cells in the presence of the cap-dependent translation inhibitor, 4E1RCat. This inhibitor disrupts interactions of eukaryotic initiation factor 4E (the cap binding protein) with initiation factors 4G and 4E-BP1 at low micromolar potencies (42). Translation of the native nanoluciferase reporter was reduced by ≥ 85% in HEK293T cells in the presence of 100 μM 4E1RCat after 4 h. We also analyzed the potential for mutants to create a uORF, given that non-canonical uORFs generally have an inhibitory effect on translation (26,27). This analysis confirmed (see Materials and Methods) that non-canonical uORF translation is unlikely either to occur or to alter translation from the primary ORF. Thus, translation of our native sequence and mutant reporters proceeds via a standard cap-dependent mechanism and from the same AUG start as the native transcript.

**Figure 5.**
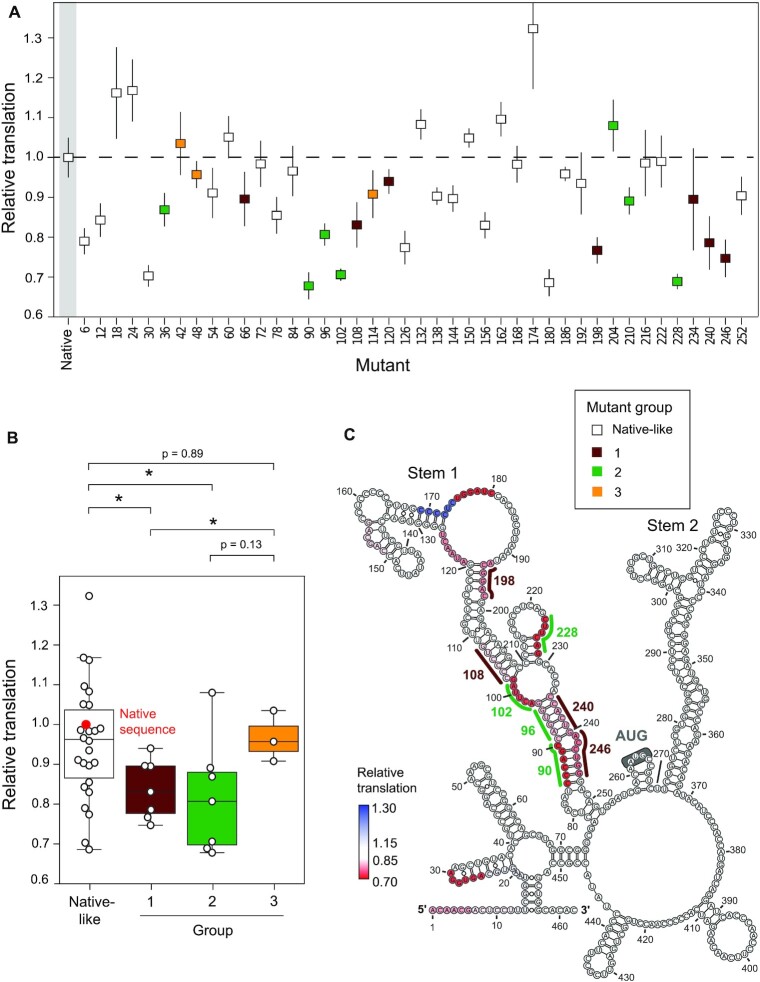
Translation depends on NM_000295.4 structure. (**A**) Translation of mutants relative to the native sequence construct, measured by dual luciferase assay. Mutants are ordered 5′ to 3′ and colored by structural group classification. Error bars show standard deviations (*n* = 6, two plasmid replicates each comprising three biological replicates). (**B**) Distribution of relative translation, as a function of structural group. Individual mutants are plotted as points; native sequence construct is shown in red. Median is shown as horizontal line; boxes show the interquartile range [IQR, from quartile 1 (Q1) to quartile 3 (Q3)]; whiskers highlight the range, Q1 – 1.5 × IQR to Q3 + 1.5 × IQR. *P ≤ 0.05 (two-tailed *t*-test). (**C**) Superposition of mutation positions that folded into non-native global structures and altered translation by ≥15% on the native sequence NM_000295.4 5′-UTR structure (based on cell-free data).

We assessed the effects of mutations on cap-dependent translation by measuring the luminescent output of nanoluciferase expressed from each mutant reporter (50). Nanoluciferase signal from each mutant was normalized to that from a constant, co-expressed control firefly luciferase plasmid. The signal ratio for each mutant (nanoluciferase/firefly) is reported relative to the native sequence NM_000295.4 5′-UTR reporter signal ratio. Most mutations either had no impact or decreased translation: 25 had no effect (<15% change translation); 14 mutants decreased translation by ≥ 15%; and only three mutants increased translation by ≥15% (Figure 5A). Mutations that modulated translation are located throughout the 5′-UTR. In some cases, adjacent mutations, such as mutants 6 and 12 compared to 18 and 24, and mutant 174 compared to 180, had notably different effects on translation (Figure 5A). Thus, the simple sequence position of mutants was not strongly predictive of translation.

We next assessed the effects on translation for each SHAPE-defined structural group. Mutants in the native-like structure group showed a large variance in their effect on translation. Although 16 native-like mutants had no effect, 6 mutants decreased and 3 mutants increased luciferase signal by 15% or more (Figure 5B). Some native-like mutants that caused significant changes in translation were in single-stranded regions of the native sequence RNA, suggesting structure has a limited role in regulating translation at these sites (Figure 5C). For example, mutants 6 and 12, which decrease translation, are located in single-stranded regions near the 5′ end of the RNA, where cap-binding translation initiation factors are likely to interact. Mutant 174, which increased translation by 32%, and mutant 180, which reduced translation by 32%, are adjacent mutations that overlap the same single-stranded loop region. These data suggest that cryptic local structural features or protein binding in these regions regulate translation of the NM_000295.4 mRNA.

Intriguingly, despite inducing global remodeling of 5′-UTR structure, none of the mutants in the three non-native structure groups significantly increased translation (Figure 5A). Group 1 and 2 mutants generally inhibited translation, whereas group 3 mutants did not impact translation (Figure 5B). Thus, global 5′-UTR RNA structure modulates translation, and relative to non-native mutants, the native structure appears optimized for translation. The group 1 and 2 mutations with the largest impact on translation (90, 96, 102, 108, 228, 240 and 246) occurred at the base of Stem 1, severely destabilizing this helix and causing the RNA to refold into non-native structure (Figures 4B and 5C). Thus, Stem 1 appears to enforce a global 5′-UTR architecture that optimizes translation.

### Start codon accessibility governs translation in 5′-UTR mutants

To further understand the mechanism through which disruption of Stem 1 downregulates translation, we more closely examined RNA refolding that occurs adjacent to the start codon.

**Figure 6.**
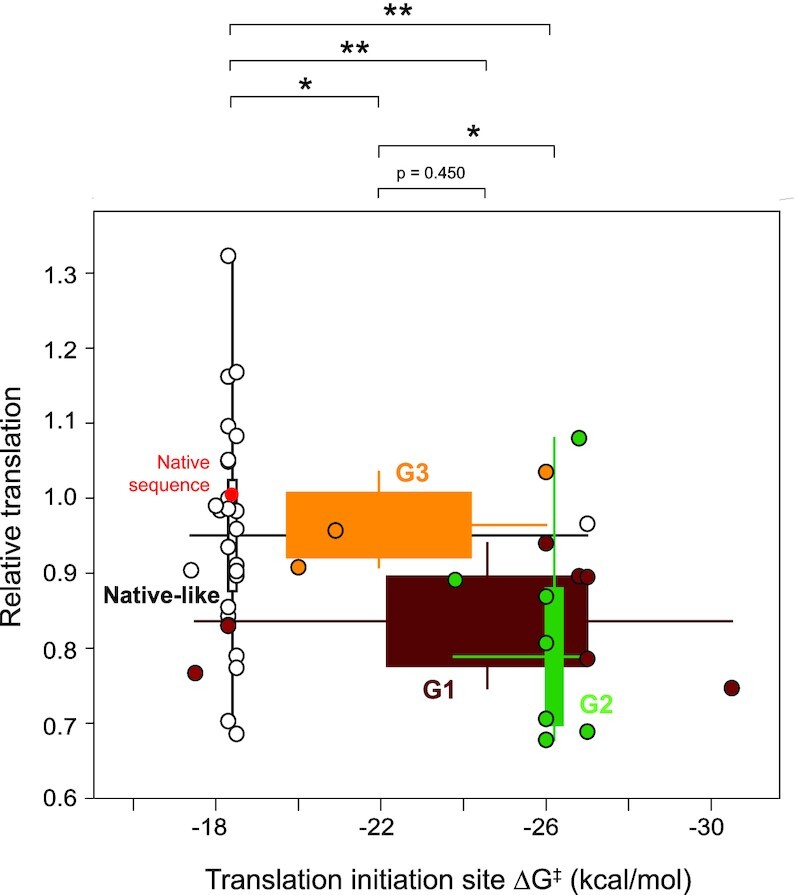
Translation is anti-correlated with structure at the translation initiation site. Relationship between translation and energetic cost of unfolding structures at the translation initiation site for each structure group. Δ*G*^‡^_unfold_ was calculated for a window of ±15 nucleotides from the adenosine of the start codon (analysis of alternative window sizes shown in Supplementary Figure S4). Relationship between translation and cost of structure unfolding (Δ*G*^‡^_unfold_) is specific to the translation initiation site (Supplementary Figure S6). Data are shown as two-dimensional box plots; in both dimensions, boxes span the IQR; whiskers extend to minimum and maximum observed values. Individual mutants are plotted as points; the native sequence is red. *P ≤ 0.05; **P≤ 0.001 (two-tailed *t*-test).

Translation initiation requires the start of the mRNA to be threaded through the 40S subunit of the ribosome in a single-stranded conformation. Thus, RNA structures must be disrupted in order to present single-stranded RNA to initiate translation (51,52). RNA structures within 13–16 nucleotides of the start codon, in both 5′ and 3′ directions, modulate translation (4,7,19,53), with 26–32 nucleotides corresponding to the estimated length of RNA that fits in the mRNA cleft for the eukaryotic ribosome (54,55). We therefore calculated the energetic penalty for the non-equilibrium unfolding (Δ*G*^‡^_unfold_) of RNA structures in a symmetric window spanning ±15 nucleotides, centered at the adenosine of the start codon for each mutant (4,19). The non-equilibrium Δ*G*^‡^_unfold_ represents the energetic cost of disrupting RNA structure without allowing RNA refolding. This non-equilibrium model yields the strongest correlation to translation efficiency relative to other RNA structure-based mechanisms (4,19). Δ*G*^‡^_unfold_ for structures formed in group 1 and 2 mutants was significantly lower (more thermodynamically stable) than structures formed by the native-like group of RNA mutants (Figure 6). These results are consistent with the increase in structure observed near the start codon across group 1 and 2 mutants due to the formation of the helix at positions 210–250 (Figure 4A, feature 5; Figure 4B, arc diagrams). Similar results were obtained when Δ*G*^‡^_unfold_ was computed for other physically reasonable ribosome footprints, indicating that this result is general and not dependent on specific unfolding window choice (Supplementary Figure S4). Mean stabilities of RNA structures formed at the translation initiation site were also higher for groups 1 and 2 than for group 3 mutants, again correlating with the lower translation of groups 1 and 2 compared to group 3. The negative-correlation between RNA structure and translation is only observed when SHAPE data are used to guide structure modeling (Supplementary Figure S5) and is not observed for windows placed 5′ or 3′ of the AUG region (Supplementary Figure S6). In sum, models generated from SHAPE data suggest that stable structure specifically around the start codon limits accessibility to the ribosomal preinitiation complex, and thereby reduces translation of mutants in groups 1 and 2.

## DISCUSSION

Our work emphasizes that RNA structures do not always negatively impact translation, as has been widely thought, but rather that structures can enhance translation in specific contexts. We investigated structure-function relationships across the 5′-UTR of the NM_000295.4 isoform of the human *SERPINA1* gene using an efficient, comprehensive mutagenesis strategy. We discovered that the 5′-UTR and the CDS each form non-interacting, independent structural entities or domains. Six-nucleotide mutations in the 5′-UTR destabilized RNA structure locally, as designed, but interestingly, most mutants nonetheless adopted a native-like global structure. Most mutations that affected (experimentally confirmed) cap-dependent translation were also those that induced large-scale refolding in the 5′-UTR. Specifically, mutations that promoted formation of non-native stable structures near the start codon inhibited translation. To our knowledge, this is the first study to comprehensively examine RNA structure-function consequences at near-nucleotide resolution across an entire 5′-UTR.

The most striking finding in our study is that most structure-destabilizing substitutions, placed comprehensively across the 5′-UTR, either did not change or reduced translation. Only three of the 42 mutations increased translation. This is surprising as the consensus model has been that reducing structure in the 5′-UTR will increase translation (7,48,56–60). SHAPE structural analysis clearly confirmed that the 5′-UUAUUA-3′ structure-destabilizing substitutions reduce local structure, as designed. However, in doing so, the destabilizing mutations sometimes promoted global RNA refolding, and thereby disrupted native 5′-UTR structures that preserved accessibility of the translation initiation site.

**Figure 7.**
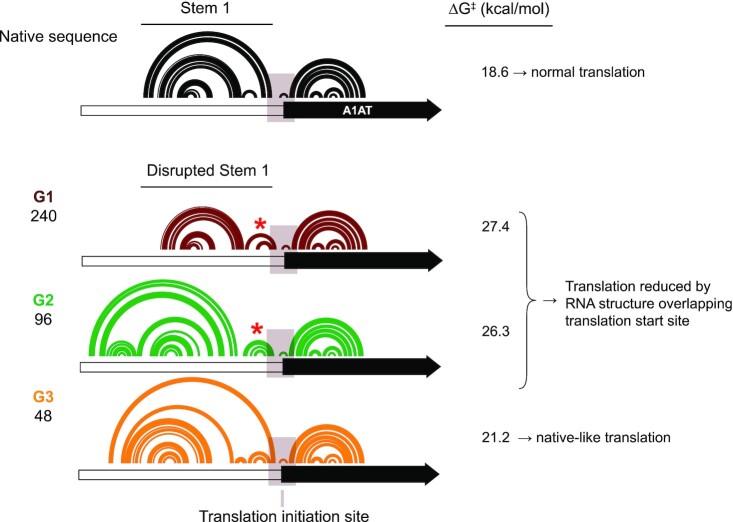
Model for regulation of translation by NM_000295.4 5′-UTR structure. Comparison of Stem 1 in the native sequence with alternative structures formed in each mutant structure group. Local RNA structure changes near the translation start site (shaded box) highlighted. Asterisks denote well-defined hairpins formed in group 1 and 2 mutants. Δ*G*^‡^_unfold_ calculated for the 30 nucleotide window emphasized with shaded box.

In the native sequence NM_000295.4 isoform, Stem 1 is a well-defined, structured motif that appears to sequester 5′-UTR RNA sequences and preserve access to the translation initiation site by the ribosome, thereby optimizing translation (Figure 7). Across the 25 mutants that form native-like structures, Stem 1 retains its fold to render NM_000295.4 resistant to translation-compromising conformational changes. However, mutations that disrupted base pairing at the base of Stem 1 induced significant changes in 5′-UTR structure. In group 1 and group 2 mutants, the 5′-UTR refolds to form a compact hairpin near the start codon, which presumably reduces accessibility of the start codon to the ribosome and decreases translation (Figure 7, red asterisk). In group 3 mutants, the global 5′-UTR RNA structure changes, but does not result in stable structure near the start codon and, consistently, these mutants did not show reduced translation relative to the native sequence construct. Intriguingly, Stem 1 appears to prevent stable RNA structures from overlapping the translation initiation site, maintaining ribosome accessibility to the start codon and optimize the NM_000295.4 isoform for translation.

There are likely other *SERPINA1* isoforms that are regulated by the Stem 1 motifs in a manner similar to NM_000295.4. Recent advances in transcription start site mapping continually identify novel transcription initiation sites and new annotations for the *SERPINA1* 5′-UTR (61–63). Several new transcription start sites have been annotated for *SERPINA1*, with shorter 5′-UTRs than NM_000295.4, but likely to retain the Stem 1 motif (61,62). These shorter transcripts likely contribute to our abundance estimates of NM_000295.4 (Supplementary Figure S1). Hence, these shorter transcripts merit direct evaluation to assess if our proposed role for Stem 1 regulation of *SERPINA1* translation occurs for other isoforms. Fundamentally, the 5′-UTR of *SERPINA1* remains an excellent model for understanding how the interplay of multiple translational regulatory mechanisms regulates gene expression in transcript- and tissue-specific ways.

In certain regions of the NM_000295.4 transcript, the primary sequence is likely important. Despite structures very similar to that of the native sequence transcript, translation of mutants 6, 12 and 30 was reduced compared to the native sequence construct. These mutations are located at the 5′-end of the mRNA, where translation initiation factors bind and facilitate ribosomal loading (64,65). Mutations 174 and 180, which are adjacent in a single-stranded RNA loop, had the largest, and opposing, effects on translation of all mutations tested (≥30% changes). We speculate that a protein binds in this region, given the lack of local structure and the substantial effects of these mutations on translation.

Our model furthers understanding of how RNA structure across a 5′-UTR influences translation. The standard model posits that the 40S subunit binds to the 5′ cap region of an mRNA and scans across the 5′-UTR in a processive manner until it detects an AUG (or perhaps a CUG (27)) start codon to form a translation-competent ribosome (58). Given that the RNA must be threaded through the 40S subunit of the ribosome, structure is generally thought to be inhibitory to this scanning process. RNA needs to be unwound for the 40S subunit both to dock at the 5′ end of the RNA and also to recognize the AUG start codon (66). Consistent with this model, early studies demonstrated that stable hairpins at either the 5′ end of an mRNA (47,57,67,68) or near or overlapping the start codon (47,51,56,57,69) inhibit translation. However, these studies evaluated very strong helices, with stabilities between 30–50 kcal/mol, and observed up to 50-fold impacts on translation (47,57). Such extended, highly stable hairpins are rarely found in native human mRNAs and do not occur in the NM_000295.4 transcript. Nevertheless, these results have been extrapolated to conclude that significant 5′-UTR secondary structures will decrease translational output (48,58,70). Exceptions to this model have been noted; for example, the *LINE* mRNA has a 900-nucleotide 5′-UTR that is 60% GC-rich and is highly translated, suggesting that secondary structure has nuanced effects on translation (71). The 261 nucleotide NM_000295.4 5′-UTR is modestly longer than the median 5′-UTR in the human transcriptome (at 218 nucleotides (18)). Translation is a complex process, and our analysis of NM_000295.4 emphasizes that the global architecture of natural sequences can enhance translation by preserving local structure at key regulatory positions in the 5′-UTR (Figure 7).

Our strategy for systematically destabilizing RNA creates a framework for understanding functional roles of complex, seemingly idiosyncratic, structures across native 5′-UTRs and identifies potential structural hotspots that support strategies for RNA-directed therapeutics in cases of pathological gene expression. The Stem 1 motif in the NM_000295.4 5′-UTR is a complex, well-defined structure that, when disrupted, reduces translation by limiting access to the translation start site by the ribosome. Mutational analysis identifies clear hot spots for expression-perturbing structure changes with the potential to both up- and down-regulate translation (Figure 5). 5′-UTRs of similar length and GC-content likely adopt similarly complex, seemingly idiosyncratic RNA structures (18,19). Our findings highlight a likely widespread mechanism whereby disrupting RNA structure induces global-refolding and blocks ribosome access to the translation start site. As experimental validation of RNA structure-function interrelationships becomes more fully embraced, we anticipate that complex and structurally distinctive (72) RNA structure-based gene regulatory elements will be broadly identified that can serve as targets for therapeutic ligand discovery.

## DATA AVAILABILITY

Sequencing reads for SHAPE-MaP structural probing data of wild-type and mutant 5′-UTRs are available in the Sequence Read Archive (SRA), Bioproject number PRJNA749882.

## Supplementary Material

gkac739_Supplemental_Files
