# Supplementary material for: Global 5′-UTR RNA structure regulates translation of a SERPINA1 mRNA
Source: Nucleic Acids Res. 2022 Sep 15;50(17):9689–704. doi: 10.1093/nar/gkac739 (PMC9508835; doi:10.1093/nar/gkac739)
Supplement: gkac739_Supplemental_Files [file gkac739_supplemental_files.zip › Dataset_S1_plasmid_sequences.docx]

Full-plasmid sequences confirmed from a de novo assembly by NGS Sequencing for all mutants. The entire mRNA region confirmed by Sanger sequencing, from 5'-UTR to the nanoluciferase poly-AAA tail for all mutants.

5'-UTR highlighted in yellow

Kozak sequence highlighted in grey

An initial portion of the *SERPINA1* coding region in pink. Rationale: This early coding sequence (CDS, 240 nucleotides) was cloned into the plasmid to analyze potential long-range structural interactions between the 5'-UTR and CDS.

NM_000295.4 upstream open reading frame (uORF) mutation (a thymine to cytosine substitution) highlighted in red. Rationale: This uORF is not thought to be active in the endogenous gene due to limited initiation site for the ribosome. We substituted the ATG to an ACG to reduce off-target translation initiation to mirror endogenous gene regulation. This mutation is highlighted in red.

Nanoluciferase Coding Sequence in green. A 29 amino acid linker (white region preceding luciferase gene) connected the early *SERPINA1* coding protein to the nanoluciferase gene.

>Wildtype_*SERPINA1*_5'-UTR Plasmid

GGCCTAACTGGCCTCAATATTGGCCATTAGCCATATTATTCATTGGTTATATAGCATAAATCAATATTGGCTATTGGCCATTGCATACGTTGTATCTATATCATAATATGTACATTTATATTGGCTCATGTCCAATATGACCGCCATGTTGGCATTGATTATTGACTAGTTATTAATAGTAATCAATTACGGGGTCATTAGTTCATAGCCCATATATGGAGTTCCGCGTTACATAACTTACGGTAAATGGCCCGCCTGGCTGACCGCCCAACGACCCCCGCCCATTGACGTCAATAATGACGTATGTTCCCATAGTAACGCCAATAGGGACTTTCCATTGACGTCAATGGGTGGAGTATTTACGGTAAACTGCCCACTTGGCAGTACATCAAGTGTATCATATGCCAAGTCCGCCCCCTATTGACGTCAATGACGGTAAATGGCCCGCCTGGCATTATGCCCAGTACATGACCTTACGGGACTTTCCTACTTGGCAGTACATCTACGTATTAGTCATCGCTATTACCATGGTGATGCGGTTTTGGCAGTACACCAATGGGCGTGGATAGCGGTTTGACTCACGGGGATTTCCAAGTCTCCACCCCATTGACGTCAATGGGAGTTTGTTTTGGCACCAAAATCAACGGGACTTTCCAAAATGTCGTAATAACCCCGCCCCGTTGACGCAAATGGGCGGTAGGCGTGTACGGTGGGAGGTCTATATAAGCAGAGCTCGTTTAGTGAACCGTCAGATCACTAGAAGCTTTATTGCGGTAGTTTATCACAGTCTCGAGACAA**C**GACTCCTTTCGGTAAGTGCAGTGGAAGCTGTACACTGCCCAGGCAAAGCGTCCGGGCAGCGTAGGCGGGCGACTCAGATCCCAGCCAGTGGACTTAGCCCCTGTTTGCTCCTCCGATAACTGGGGTGACCTTGGTTAATATTCACCAGCAGCCTCCCCCGTTGCCCCTCTGGATCCACTGCTTAAATACGGACGAGGACAGGGCCCTGTCTCCTCAGCTTCAGGCACCACCACTGACCTGGGACAGTGAATCGACAATGCCGTCTTCTGTCTCGTGGGGCATCCTCCTGCTGGCAGGCCTGTGCTGCCTGGTCCCTGTCTCCCTGGCTGAGGATCCCCAGGGAGATGCTGCCCAGAAGACAGATACATCCCACCATGATCAGGATCACCCAACCTTCAACAAGATCACCCCCAACCTGGCTGAGTTCGCCTTCAGCCTATACCGCCAGCTGGCACACCAGTCCAACAGCACCAATATCTTCTTCTCCCCAGTGAGTGGTACCTTGCTAGCACGAGCGGCCGCTAAATTGCTAACGCAGTCAGTGGGCCTCGGCGGCCAAGCTTGGCAATCCGGTACTGTTGGTGTCTTCACACTCGAAGATTTCGTTGGGGACTGGCGACAGACAGCCGGCTACAACCTGGACCAAGTCCTTGAACAGGGAGGTGTGTCCAGTTTGTTTCAGAATCTCGGGGTGTCCGTAACTCCGATCCAAAGGATTGTCCTGAGCGGTGAAAATGGGCTGAAGATCGACATCCATGTCATCATCCCGTATGAAGGTCTGAGCGGCGACCAAATGGGCCAGATCGAAAAAATTTTTAAGGTGGTGTACCCTGTGGATGATCATCACTTTAAGGTGATCCTGCACTATGGCACACTGGTAATCGACGGGGTTACGCCGAACATGATCGACTATTTCGGACGGCCGTATGAAGGCATCGCCGTGTTCGACGGCAAAAAGATCACTGTAACAGGGACCCTGTGGAACGGCAACAAAATTATCGACGAGCGCCTGATCAACCCCGACGGCTCCCTGCTGTTCCGAGTAACCATCAACGGAGTGACCGGCTGGCGGCTGTGCGAACGCATTCTGGCGAATGCTCGCATCAACGTCTAAGGCCGCGACTCTAGAGTCGGGGCGGCCGGCCGCTTCGAGCAGACATGATAAGATACATTGATGAGTTTGGACAAACCACAACTAGAATGCAGTGAAAAAAATGCTTTATTTGTGAAATTTGTGATGCTATTGCTTTATTTGTAACCATTATAAGCTGCAATAAACAAGTTAACAACAACAATTGCATTCATTTTATGTTTCAGGTTCAGGGGGAGGTGTGGGAGGTTTTTTAAAGCAAGTAAAACCTCTACAAATGTGGTAAAATCGATAAGGATCCGTTTGCGTATTGGGCGCTCTTCCGCTGCGCTCTTCCGCTTCCTCGCTCACTGACTCGCTGCGCTCGGTCGTTCGGCTGCGGCGAGCGGTATCAGCTCACTCAAAGGCGGTAATACGGTTATCCACAGAATCAGGGGATAACGCAGGAAAGAACATGTGAGCAAAAGGCCAGCAAAAGGCCAGGAACCGTAAAAAGGCCGCGTTGCTGGCGTTTTTCCATAGGCTCCGCCCCCCTGACGAGCATCACAAAAATCGACGCTCAAGTCAGAGGTGGCGAAACCCGACAGGACTATAAAGATACCAGGCGTTTCCCCCTGGAAGCTCCCTCGTGCGCTCTCCTGTTCCGACCCTGCCGCTTACCGGATACCTGTCCGCCTTTCTCCCTTCGGGAAGCGTGGCGCTTTCTCATAGCTCACGCTGTAGGTATCTCAGTTCGGTGTAGGTCGTTCGCTCCAAGCTGGGCTGTGTGCACGAACCCCCCGTTCAGCCCGACCGCTGCGCCTTATCCGGTAACTATCGTCTTGAGTCCAACCCGGTAAGACACGACTTATCGCCACTGGCAGCAGCCACTGGTAACAGGATTAGCAGAGCGAGGTATGTAGGCGGTGCTACAGAGTTCTTGAAGTGGTGGCCTAACTACGGCTACACTAGAAGAACAGTATTTGGTATCTGCGCTCTGCTGAAGCCAGTTACCTTCGGAAAAAGAGTTGGTAGCTCTTGATCCGGCAAACAAACCACCGCTGGTAGCGGTGGTTTTTTTGTTTGCAAGCAGCAGATTACGCGCAGAAAAAAAGGATCTCAAGAAGATCCTTTGATCTTTTCTACGGGGTCTGACGCTCAGTGGAACGAAAACTCACGTTAAGGGATTTTGGTCATGAGATTATCAAAAAGGATCTTCACCTAGATCCTTTTAAATTAAAAATGAAGTTTTAAATCAATCTAAAGTATATATGAGTAAACTTGGTCTGACAGCGGCCGCAAATGCTAAACCACTGCAGTGGTTACCAGTGCTTGATCAGTGAGGCACCGATCTCAGCGATCTGCCTATTTCGTTCGTCCATAGTGGCCTGACTCCCCGTCGTGTAGATCACTACGATTCGTGAGGGCTTACCATCAGGCCCCAGCGCAGCAATGATGCCGCGAGAGCCGCGTTCACCGGCCCCCGATTTGTCAGCAATGAACCAGCCAGCAGGGAGGGCCGAGCGAAGAAGTGGTCCTGCTACTTTGTCCGCCTCCATCCAGTCTATGAGCTGCTGTCGTGATGCTAGAGTAAGAAGTTCGCCAGTGAGTAGTTTCCGAAGAGTTGTGGCCATTGCTACTGGCATCGTGGTATCACGCTCGTCGTTCGGTATGGCTTCGTTCAACTCTGGTTCCCAGCGGTCAAGCCGGGTCACATGATCACCCATATTATGAAGAAATGCAGTCAGCTCCTTAGGGCCTCCGATCGTTGTCAGAAGTAAGTTGGCCGCGGTGTTGTCGCTCATGGTAATGGCAGCACTACACAATTCTCTTACCGTCATGCCATCCGTAAGATGCTTTTCCGTGACCGGCGAGTACTCAACCAAGTCGTTTTGTGAGTAGTGTATACGGCGACCAAGCTGCTCTTGCCCGGCGTCTATACGGGACAACACCGCGCCACATAGCAGTACTTTGAAAGTGCTCATCATCGGGAATCGTTCTTCGGGGCGGAAAGACTCAAGGATCTTGCCGCTATTGAGATCCAGTTCGATATAGCCCACTCTTGCACCCAGTTGATCTTCAGCATCTTTTACTTTCACCAGCGTTTCGGGGTGTGCAAAAACAGGCAAGCAAAATGCCGCAAAGAAGGGAATGAGTGCGACACGAAAATGTTGGATGCTCATACTCGTCCTTTTTCAATATTATTGAAGCATTTATCAGGGTTACTAGTACGTCTCTCAAGGATAAGTAAGTAATATTAAGGTACGGGAGGTATTGGACAGGCCGCAATAAAATATCTTTATTTTCATTACATCTGTGTGTTGGTTTTTTGTGTGAATCGATAGTACTAACATACGCTCTCCATCAAAACAAAACGAAACAAAACAAACTAGCAAAATAGGCTGTCCCCAGTGCAAGTGCAGGTGCCAGAACATTTCTCT

5'-UTR mutation inserts:

Mutants 5'-UTRs were inserted by homologous recombination (Twist Bioscience) to replace the wildtype 5'-UTR (yellow region) above without the need to introduce or delete any additional nucleotides:

The tiling six-nucleotide substitution is CAPITALIZED. In mutant 174, the TTATTA sequence was converted to a TTATTT sequence to avoid introducing an unintended stop codon.

Kozak sequence highlighted in grey

| Mutant | Sequence |
| --- | --- |
| 6 | TTATTAactcctttcggtaagtgcagtggaagctgtacactgcccaggcaaagcgtccgggcagcgtaggcgggcgactcagatcccagccagtggacttagcccctgtttgctcctccgataactggggtgaccttggttaatattcaccagcagcctcccccgttgcccctctggatccactgcttaaatacggacgaggacagggccctgtctcctcagcttcaggcaccaccactgacctgggacagtgaaTCGACAATGC |
| 12 | acaacgTTATTAttcggtaagtgcagtggaagctgtacactgcccaggcaaagcgtccgggcagcgtaggcgggcgactcagatcccagccagtggacttagcccctgtttgctcctccgataactggggtgaccttggttaatattcaccagcagcctcccccgttgcccctctggatccactgcttaaatacggacgaggacagggccctgtctcctcagcttcaggcaccaccactgacctgggacagtgaaTCGACAATGC |
| 18 | acaacgactcctTTATTAaagtgcagtggaagctgtacactgcccaggcaaagcgtccgggcagcgtaggcgggcgactcagatcccagccagtggacttagcccctgtttgctcctccgataactggggtgaccttggttaatattcaccagcagcctcccccgttgcccctctggatccactgcttaaatacggacgaggacagggccctgtctcctcagcttcaggcaccaccactgacctgggacagtgaaTCGACAATGC |
| 24 | acaacgactcctttcggtTTATTAagtggaagctgtacactgcccaggcaaagcgtccgggcagcgtaggcgggcgactcagatcccagccagtggacttagcccctgtttgctcctccgataactggggtgaccttggttaatattcaccagcagcctcccccgttgcccctctggatccactgcttaaatacggacgaggacagggccctgtctcctcagcttcaggcaccaccactgacctgggacagtgaaTCGACAATGC |
| 30 | acaacgactcctttcggtaagtgcTTATTAagctgtacactgcccaggcaaagcgtccgggcagcgtaggcgggcgactcagatcccagccagtggacttagcccctgtttgctcctccgataactggggtgaccttggttaatattcaccagcagcctcccccgttgcccctctggatccactgcttaaatacggacgaggacagggccctgtctcctcagcttcaggcaccaccactgacctgggacagtgaaTCGACAATGC |
| 36 | acaacgactcctttcggtaagtgcagtggaTTATTAacactgcccaggcaaagcgtccgggcagcgtaggcgggcgactcagatcccagccagtggacttagcccctgtttgctcctccgataactggggtgaccttggttaatattcaccagcagcctcccccgttgcccctctggatccactgcttaaatacggacgaggacagggccctgtctcctcagcttcaggcaccaccactgacctgggacagtgaaTCGACAATGC |
| 42 | acaacgactcctttcggtaagtgcagtggaagctgtTTATTAcccaggcaaagcgtccgggcagcgtaggcgggcgactcagatcccagccagtggacttagcccctgtttgctcctccgataactggggtgaccttggttaatattcaccagcagcctcccccgttgcccctctggatccactgcttaaatacggacgaggacagggccctgtctcctcagcttcaggcaccaccactgacctgggacagtgaaTCGACAATGC |
| 48 | acaacgactcctttcggtaagtgcagtggaagctgtacactgTTATTAcaaagcgtccgggcagcgtaggcgggcgactcagatcccagccagtggacttagcccctgtttgctcctccgataactggggtgaccttggttaatattcaccagcagcctcccccgttgcccctctggatccactgcttaaatacggacgaggacagggccctgtctcctcagcttcaggcaccaccactgacctgggacagtgaaTCGACAATGC |
| 54 | acaacgactcctttcggtaagtgcagtggaagctgtacactgcccaggTTATTAgtccgggcagcgtaggcgggcgactcagatcccagccagtggacttagcccctgtttgctcctccgataactggggtgaccttggttaatattcaccagcagcctcccccgttgcccctctggatccactgcttaaatacggacgaggacagggccctgtctcctcagcttcaggcaccaccactgacctgggacagtgaaTCGACAATGC |
| 60 | acaacgactcctttcggtaagtgcagtggaagctgtacactgcccaggcaaagcTTATTAgcagcgtaggcgggcgactcagatcccagccagtggacttagcccctgtttgctcctccgataactggggtgaccttggttaatattcaccagcagcctcccccgttgcccctctggatccactgcttaaatacggacgaggacagggccctgtctcctcagcttcaggcaccaccactgacctgggacagtgaaTCGACAATGC |
| 66 | acaacgactcctttcggtaagtgcagtggaagctgtacactgcccaggcaaagcgtccggTTATTAtaggcgggcgactcagatcccagccagtggacttagcccctgtttgctcctccgataactggggtgaccttggttaatattcaccagcagcctcccccgttgcccctctggatccactgcttaaatacggacgaggacagggccctgtctcctcagcttcaggcaccaccactgacctgggacagtgaaTCGACAATGC |
| 72 | acaacgactcctttcggtaagtgcagtggaagctgtacactgcccaggcaaagcgtccgggcagcgTTATTAggcgactcagatcccagccagtggacttagcccctgtttgctcctccgataactggggtgaccttggttaatattcaccagcagcctcccccgttgcccctctggatccactgcttaaatacggacgaggacagggccctgtctcctcagcttcaggcaccaccactgacctgggacagtgaaTCGACAATGC |
| 78 | acaacgactcctttcggtaagtgcagtggaagctgtacactgcccaggcaaagcgtccgggcagcgtaggcgTTATTAtcagatcccagccagtggacttagcccctgtttgctcctccgataactggggtgaccttggttaatattcaccagcagcctcccccgttgcccctctggatccactgcttaaatacggacgaggacagggccctgtctcctcagcttcaggcaccaccactgacctgggacagtgaaTCGACAATGC |
| 84 | acaacgactcctttcggtaagtgcagtggaagctgtacactgcccaggcaaagcgtccgggcagcgtaggcgggcgacTTATTAcccagccagtggacttagcccctgtttgctcctccgataactggggtgaccttggttaatattcaccagcagcctcccccgttgcccctctggatccactgcttaaatacggacgaggacagggccctgtctcctcagcttcaggcaccaccactgacctgggacagtgaaTCGACAATGC |
| 90 | acaacgactcctttcggtaagtgcagtggaagctgtacactgcccaggcaaagcgtccgggcagcgtaggcgggcgactcagatTTATTAcagtggacttagcccctgtttgctcctccgataactggggtgaccttggttaatattcaccagcagcctcccccgttgcccctctggatccactgcttaaatacggacgaggacagggccctgtctcctcagcttcaggcaccaccactgacctgggacagtgaaTCGACAATGC |
| 96 | acaacgactcctttcggtaagtgcagtggaagctgtacactgcccaggcaaagcgtccgggcagcgtaggcgggcgactcagatcccagcTTATTAacttagcccctgtttgctcctccgataactggggtgaccttggttaatattcaccagcagcctcccccgttgcccctctggatccactgcttaaatacggacgaggacagggccctgtctcctcagcttcaggcaccaccactgacctgggacagtgaaTCGACAATGC |
| 102 | acaacgactcctttcggtaagtgcagtggaagctgtacactgcccaggcaaagcgtccgggcagcgtaggcgggcgactcagatcccagccagtggTTATTAcccctgtttgctcctccgataactggggtgaccttggttaatattcaccagcagcctcccccgttgcccctctggatccactgcttaaatacggacgaggacagggccctgtctcctcagcttcaggcaccaccactgacctgggacagtgaaTCGACAATGC |
| 108 | acaacgactcctttcggtaagtgcagtggaagctgtacactgcccaggcaaagcgtccgggcagcgtaggcgggcgactcagatcccagccagtggacttagTTATTAtttgctcctccgataactggggtgaccttggttaatattcaccagcagcctcccccgttgcccctctggatccactgcttaaatacggacgaggacagggccctgtctcctcagcttcaggcaccaccactgacctgggacagtgaaTCGACAATGC |
| 114 | acaacgactcctttcggtaagtgcagtggaagctgtacactgcccaggcaaagcgtccgggcagcgtaggcgggcgactcagatcccagccagtggacttagcccctgTTATTAcctccgataactggggtgaccttggttaatattcaccagcagcctcccccgttgcccctctggatccactgcttaaatacggacgaggacagggccctgtctcctcagcttcaggcaccaccactgacctgggacagtgaaTCGACAATGC |
| 120 | acaacgactcctttcggtaagtgcagtggaagctgtacactgcccaggcaaagcgtccgggcagcgtaggcgggcgactcagatcccagccagtggacttagcccctgtttgctTTATTAataactggggtgaccttggttaatattcaccagcagcctcccccgttgcccctctggatccactgcttaaatacggacgaggacagggccctgtctcctcagcttcaggcaccaccactgacctgggacagtgaaTCGACAATGC |
| 126 | acaacgactcctttcggtaagtgcagtggaagctgtacactgcccaggcaaagcgtccgggcagcgtaggcgggcgactcagatcccagccagtggacttagcccctgtttgctcctccgTTATTAggggtgaccttggttaatattcaccagcagcctcccccgttgcccctctggatccactgcttaaatacggacgaggacagggccctgtctcctcagcttcaggcaccaccactgacctgggacagtgaaTCGACAATGC |
| 132 | acaacgactcctttcggtaagtgcagtggaagctgtacactgcccaggcaaagcgtccgggcagcgtaggcgggcgactcagatcccagccagtggacttagcccctgtttgctcctccgataactTTATTAaccttggttaatattcaccagcagcctcccccgttgcccctctggatccactgcttaaatacggacgaggacagggccctgtctcctcagcttcaggcaccaccactgacctgggacagtgaaTCGACAATGC |
| 138 | acaacgactcctttcggtaagtgcagtggaagctgtacactgcccaggcaaagcgtccgggcagcgtaggcgggcgactcagatcccagccagtggacttagcccctgtttgctcctccgataactggggtgTTATTAgttaatattcaccagcagcctcccccgttgcccctctggatccactgcttaaatacggacgaggacagggccctgtctcctcagcttcaggcaccaccactgacctgggacagtgaaTCGACAATGC |
| 144 | acaacgactcctttcggtaagtgcagtggaagctgtacactgcccaggcaaagcgtccgggcagcgtaggcgggcgactcagatcccagccagtggacttagcccctgtttgctcctccgataactggggtgaccttgTTATTAattcaccagcagcctcccccgttgcccctctggatccactgcttaaatacggacgaggacagggccctgtctcctcagcttcaggcaccaccactgacctgggacagtgaaTCGACAATGC |
| 150 | acaacgactcctttcggtaagtgcagtggaagctgtacactgcccaggcaaagcgtccgggcagcgtaggcgggcgactcagatcccagccagtggacttagcccctgtttgctcctccgataactggggtgaccttggttaatTTATTAcagcagcctcccccgttgcccctctggatccactgcttaaatacggacgaggacagggccctgtctcctcagcttcaggcaccaccactgacctgggacagtgaaTCGACAATGC |
| 156 | acaacgactcctttcggtaagtgcagtggaagctgtacactgcccaggcaaagcgtccgggcagcgtaggcgggcgactcagatcccagccagtggacttagcccctgtttgctcctccgataactggggtgaccttggttaatattcacTTATTAcctcccccgttgcccctctggatccactgcttaaatacggacgaggacagggccctgtctcctcagcttcaggcaccaccactgacctgggacagtgaaTCGACAATGC |
| 162 | acaacgactcctttcggtaagtgcagtggaagctgtacactgcccaggcaaagcgtccgggcagcgtaggcgggcgactcagatcccagccagtggacttagcccctgtttgctcctccgataactggggtgaccttggttaatattcaccagcagTTATTAccgttgcccctctggatccactgcttaaatacggacgaggacagggccctgtctcctcagcttcaggcaccaccactgacctgggacagtgaaTCGACAATGC |
| 168 | acaacgactcctttcggtaagtgcagtggaagctgtacactgcccaggcaaagcgtccgggcagcgtaggcgggcgactcagatcccagccagtggacttagcccctgtttgctcctccgataactggggtgaccttggttaatattcaccagcagcctcccTTATTAcccctctggatccactgcttaaatacggacgaggacagggccctgtctcctcagcttcaggcaccaccactgacctgggacagtgaaTCGACAATGC |
| 174* | acaacgactcctttcggtaagtgcagtggaagctgtacactgcccaggcaaagcgtccgggcagcgtaggcgggcgactcagatcccagccagtggacttagcccctgtttgctcctccgataactggggtgaccttggttaatattcaccagcagcctcccccgttgTTATTTtggatccactgcttaaatacggacgaggacagggccctgtctcctcagcttcaggcaccaccactgacctgggacagtgaaTCGACAATGC |
| 180 | acaacgactcctttcggtaagtgcagtggaagctgtacactgcccaggcaaagcgtccgggcagcgtaggcgggcgactcagatcccagccagtggacttagcccctgtttgctcctccgataactggggtgaccttggttaatattcaccagcagcctcccccgttgcccctcTTATTAcactgcttaaatacggacgaggacagggccctgtctcctcagcttcaggcaccaccactgacctgggacagtgaaTCGACAATGC |
| 186 | acaacgactcctttcggtaagtgcagtggaagctgtacactgcccaggcaaagcgtccgggcagcgtaggcgggcgactcagatcccagccagtggacttagcccctgtttgctcctccgataactggggtgaccttggttaatattcaccagcagcctcccccgttgcccctctggatcTTATTAttaaatacggacgaggacagggccctgtctcctcagcttcaggcaccaccactgacctgggacagtgaaTCGACAATGC |
| 192 | acaacgactcctttcggtaagtgcagtggaagctgtacactgcccaggcaaagcgtccgggcagcgtaggcgggcgactcagatcccagccagtggacttagcccctgtttgctcctccgataactggggtgaccttggttaatattcaccagcagcctcccccgttgcccctctggatccactgcTTATTAacggacgaggacagggccctgtctcctcagcttcaggcaccaccactgacctgggacagtgaaTCGACAATGC |
| 198 | acaacgactcctttcggtaagtgcagtggaagctgtacactgcccaggcaaagcgtccgggcagcgtaggcgggcgactcagatcccagccagtggacttagcccctgtttgctcctccgataactggggtgaccttggttaatattcaccagcagcctcccccgttgcccctctggatccactgcttaaatTTATTAgaggacagggccctgtctcctcagcttcaggcaccaccactgacctgggacagtgaaTCGACAATGC |
| 204 | acaacgactcctttcggtaagtgcagtggaagctgtacactgcccaggcaaagcgtccgggcagcgtaggcgggcgactcagatcccagccagtggacttagcccctgtttgctcctccgataactggggtgaccttggttaatattcaccagcagcctcccccgttgcccctctggatccactgcttaaatacggacTTATTAagggccctgtctcctcagcttcaggcaccaccactgacctgggacagtgaaTCGACAATGC |
| 210 | acaacgactcctttcggtaagtgcagtggaagctgtacactgcccaggcaaagcgtccgggcagcgtaggcgggcgactcagatcccagccagtggacttagcccctgtttgctcctccgataactggggtgaccttggttaatattcaccagcagcctcccccgttgcccctctggatccactgcttaaatacggacgaggacTTATTActgtctcctcagcttcaggcaccaccactgacctgggacagtgaaTCGACAATGC |
| 216 | acaacgactcctttcggtaagtgcagtggaagctgtacactgcccaggcaaagcgtccgggcagcgtaggcgggcgactcagatcccagccagtggacttagcccctgtttgctcctccgataactggggtgaccttggttaatattcaccagcagcctcccccgttgcccctctggatccactgcttaaatacggacgaggacagggccTTATTAcctcagcttcaggcaccaccactgacctgggacagtgaaTCGACAATGC |
| 222 | acaacgactcctttcggtaagtgcagtggaagctgtacactgcccaggcaaagcgtccgggcagcgtaggcgggcgactcagatcccagccagtggacttagcccctgtttgctcctccgataactggggtgaccttggttaatattcaccagcagcctcccccgttgcccctctggatccactgcttaaatacggacgaggacagggccctgtctTTATTActtcaggcaccaccactgacctgggacagtgaaTCGACAATGC |
| 228 | acaacgactcctttcggtaagtgcagtggaagctgtacactgcccaggcaaagcgtccgggcagcgtaggcgggcgactcagatcccagccagtggacttagcccctgtttgctcctccgataactggggtgaccttggttaatattcaccagcagcctcccccgttgcccctctggatccactgcttaaatacggacgaggacagggccctgtctcctcagTTATTAgcaccaccactgacctgggacagtgaaTCGACAATGC |
| 234 | acaacgactcctttcggtaagtgcagtggaagctgtacactgcccaggcaaagcgtccgggcagcgtaggcgggcgactcagatcccagccagtggacttagcccctgtttgctcctccgataactggggtgaccttggttaatattcaccagcagcctcccccgttgcccctctggatccactgcttaaatacggacgaggacagggccctgtctcctcagcttcagTTATTAccactgacctgggacagtgaaTCGACAATGC |
| 240 | acaacgactcctttcggtaagtgcagtggaagctgtacactgcccaggcaaagcgtccgggcagcgtaggcgggcgactcagatcccagccagtggacttagcccctgtttgctcctccgataactggggtgaccttggttaatattcaccagcagcctcccccgttgcccctctggatccactgcttaaatacggacgaggacagggccctgtctcctcagcttcaggcaccaTTATTAacctgggacagtgaaTCGACAATGC |
| 246 | acaacgactcctttcggtaagtgcagtggaagctgtacactgcccaggcaaagcgtccgggcagcgtaggcgggcgactcagatcccagccagtggacttagcccctgtttgctcctccgataactggggtgaccttggttaatattcaccagcagcctcccccgttgcccctctggatccactgcttaaatacggacgaggacagggccctgtctcctcagcttcaggcaccaccactgTTATTAgacagtgaaTCGACAATGC |
| 252 | acaacgactcctttcggtaagtgcagtggaagctgtacactgcccaggcaaagcgtccgggcagcgtaggcgggcgactcagatcccagccagtggacttagcccctgtttgctcctccgataactggggtgaccttggttaatattcaccagcagcctcccccgttgcccctctggatccactgcttaaatacggacgaggacagggccctgtctcctcagcttcaggcaccaccactgacctggTTATTAgaaTCGACAATGC |
