# Supplementary material for: Global 5′-UTR RNA structure regulates translation of a SERPINA1 mRNA
Source: Nucleic Acids Res. 2022 Sep 15;50(17):9689–704. doi: 10.1093/nar/gkac739 (PMC9508835; doi:10.1093/nar/gkac739)
Supplement: gkac739_Supplemental_Files [file gkac739_supplemental_files.zip › grayeski_SI_081122.pdf]

Supporting Information for:

Global 5'-UTR RNA structure regulates translation of a *SERPINA1* mRNA

Philip J. Grayeski, Chase A. Weidmann, Jayashree Kumar, Lela Lackey, Anthony M. Mustoe, Steven Busan, Alain Laederach* and Kevin M. Weeks*

* correspondence: weeks@unc.edu, alain@unc.edu

Supporting tables (6), figures (6), and description of datasets (2)

Table S1. Correlations of the ratio in mutational profiling rates from 5NIA and DMSO-treated samples of 5'-UTR and N-terminal CDS (240 nts) structure for endogenous NM_000295.4 and plasmid-based reporter under in-cell and cell-free conditions.

Pearson correlation		Endogenous		Plasmid	
		In-cell	Cell-free	In-cell	Cell-free
Endogenous	In-cell	0.407	0.418	0.550	0.367
	Cell-free		0.717	0.681	0.754
Plasmid	In-cell			0.871	0.782
	Cell-free				0.974

[a] Pearson correlation coefficients calculated from ratio of ln(rateMod/rateUnt), where rateMod and rateUnt refer to mutation profiling rates in 5NIA-modified and untreated controls for positions 56-462, respectively. Yellow boxes report correlations between biological replicates.

Table S2. Similarities between modeled 5'-UTR and N-terminal CDS structures for endogenous NM_000295.4 and plasmid-based reporter under in-cell and cell-free conditions.

**Stem 1 | Stem 2** (Full 5'-UTR and N-terminal CDS) Arithmetic mean (%) [a]		Endogenous		Plasmid	
		In-cell	Cell-free	In-cell	Cell-free
Endogenous	In-cell	100 | 100 (100)	83 | 34 (57)	81 | 87 (65)	78 | 34 (50)
	Cell-free		100 | 100 (100)	92 | 41 (63)	95 | 97 (89)
Plasmid	In-cell			100 | 100 (100)	87 | 41 (72)
	Cell-free				100 | 100 (100)

[a] Percent arithmetic mean is (mean of sens and ppv) × 100; sens and ppv were calculated between pairs with predicted probability >0.5. Yellow boxes are percent arithmetic means calculated between two biological replicates.

Table S3. 5'-UTR and N-terminal CDS SHAPE reactivity correlation and similarity for structure models obtained from biological replicates

Construct	SHAPE correlation [a]	Modeling similarity [b]		
		sens	ppv	Arithmetic mean (%)
Native sequence	0.987	97	96	96
6	0.993	98	98	98
12	0.984	100	100	100
18	0.974	97	96	96
24	0.992	100	100	100
30	0.994	100	100	100
36	0.968	97	97	97
42	0.992	100	100	100
48	0.990	95	96	95
54	0.969	97	98	98
60	0.990	100	100	100
66	0.991	99	96	98
72	0.964	84	86	85
78	0.993	97	97	97
84	0.992	79	84	82
90	0.983	84	85	85
96	0.991	86	88	87
102	0.988	93	91	92
108	0.969	88	83	85
114	0.972	82	84	83
120	0.992	96	98	97
126	0.961	98	98	98
132	0.991	94	98	96
138	0.991	99	98	99
144	0.964	91	94	92
150	0.993	100	99	100
156	0.990	100	100	100

162	0.968	97	95	96
168	0.991	98	99	99
174	0.990	98	98	98
180	0.953	95	97	96
186	0.992	98	98	98
192	0.992	99	98	99
198	0.963	68	70	69
204	0.993	98	97	98
210	0.990	90	91	90
216	0.964	97	97	97
222	0.991	98	98	98
228	0.991	91	89	90
234	0.972	82	89	85
240	0.994	95	96	96
246	0.992	83	88	85
252	0.966	97	96	97

[a] Pearson correlation coefficients for cell-free SHAPE reactivity of 5'-UTR and N-terminal CDS between two biological replicates for each construct.

[b] Values of sens and ppv were calculated from pairing probabilities (cut-off of 0.1). Percent arithmetic means is the mean of sens and ppv.

Table S4. Comparison of structural models obtained with and without SHAPE chemical probing data [a]

Construct	sens	ppv	Arithmetic mean (%)
Native sequence	75	75	75
6	76	75	76
12	78	72	75
18	78	74	76
24	71	68	69
30	73	71	72
36	19	21	20
42	4	4	4
48	13	14	14
54	75	74	74
60	64	60	62
66	56	53	54
72	70	69	70
78	66	54	60
84	61	55	58
90	39	36	37
96	28	33	30
102	31	33	32
108	55	42	48
114	50	52	51
120	46	48	47
126	79	75	77
132	97	93	95
138	90	83	86
144	62	63	63
150	76	68	72
156	82	80	81
162	81	70	75
168	90	93	92
174	68	66	67

180	91	96	93
186	78	70	74
192	75	71	73
198	54	44	49
204	33	34	34
210	38	46	42
216	73	70	72
222	75	70	73
228	32	27	29
234	45	40	42
240	45	48	46
246	53	52	52
252	46	49	48

[a] Parameters compare structural models generated with and without using SHAPE chemical probing data. sens and ppv were calculated from pairing probabilities (cut-off of 0.1).

Table S5. Comparison of a single mutant structure model to its biological replicate and all 41 other mutants

Mutant	Replicate similarity (%) [a]	Most similar mutant [b]	Similarity (%) [c]
6	98		
12	100		
18	96	126	97
24	100		
30	100		
36	97		
42	100		
48	95		
54	98		
60	100		
66	98		
72	85	186	90
78	97		
84	82	222	84
90	85		
96	87		
102	92		
108	85		
114	83		
120	97		
126	98	192	99
132	96		
138	99		
144	92		
150	100		
156	100		
162	96	192	97
168	99		
174	98		
180	95	162	96

186	98		
192	99		
198	69	108	85
204	98		
210	90		
216	97		
222	98		
228	90		
234	85		
240	96		
246	85		
252	97		

[a] Similarity between structure models obtained from biological replicates. Similarity was calculated as arithmetic means of sens and ppv calculated from pairing probabilities (cut-off of 0.1).

[b] For each of the 42 mutants, a single structure model of a given mutant was compared against its biological replicate and the other 41 mutant structures to assess if a given mutant structure is distinct from the other 41 mutants. 35 out of all 42 mutants were most similar to their own biological replicate, but seven mutants were more similar to one of the other 41 mutants than their own biological replicate. In these seven instances, the mutant that was most similar to the single structure model is listed. For example, a single structure model for mutant 18 was most similar to mutant 126, even compared to the mutant 18 biological replicate structure model. Additionally, in those seven instances, the most similar mutant was also in the same structure group, as determined by *k*-means clustering. This result emphasizes that the structure groups identified by *k*-means clustering are both distinct from one another and exhibit high similarity within groups.

[c] Similarity of the single mutant structure compared to the non-replicate mutant structure model with highest similarity. For example, mutant 18 was 97% similar to mutant 126 compared to 96% to its biological replicate, and thus, a percentage is listed for the non-replicate mutant model with highest similarity.

Table S6. Oligonucleotides used for inverse PCR and SHAPE-MaP experiments

Primer Function	Name	Sequence
Remove ATG site for nanoluciferase coding gene	Inverse ATG F1	GTCTTCACACTCGAAGATTTCGTTGGG
	Inverse ATG R1	TTTACCAACAGTACCGGATTGCC
Substitute ATG site in SERPINA1 uORF for ACG sequence	Inverse uORF F1	CTTTCGGTAAGTGCAGTGGAAG
	Inverse uORF R1M	GAGTCGTTGTCTCGAGACTGTG
Removal of PEST sequence attached to nanoluciferase	pPEST F1	CGCTTCGAGCAGACATG
	pPEST R1	CGACTCTAGAGTCGCGG
Endogenous SERPINA1 RT Primer	RT295 Tail 4	CCCTACACGACGCTCTTCCGATCTNNNNNNNNNNNAAGAAGATATTGGTGCTGTTGGAC
Endogenous SERPINA1 Step1 PCR library generation	295 Step1 PCR Forward	GACTGGAGTTCAGACGTGTGCTCTTCCGATCTNNNNNNACTGCCCAGGCAAAGCG
	Step1 Reverse	CCCTACACGACGCTCTTCC
Plasmid SERPINA1 RT Primer	RT295 Tail 4	CCCTACACGACGCTCTTCCGATCTNNNNNNNNNNNAAGAAGATATTGGTGCTGTTGGAC
Plasmid SERPINA1 Step1 PCR library generation	Plasmid Step1 PCR Forward	GACTGGAGTTCAGACGTGTGCTCTTCCGATCTNNNNNNCGGTAGTTTATCACAGTCTCGAG
	Step1 Reverse	CCCTACACGACGCTCTTCC

Legends

Figure S1. Definition and expression of NM_000295.4. (A) Schematic of the 11 *SERPINA1* splice isoforms (chr14: 94,376,747-94,390,692). Exons shown to scale. Introns (thin grey lines) are shortened, as shown with breaks. Two versions of the NM_000295 transcript, NM_000295.4 and NM_000295.5, are highlighted. (B) Zoomed-in schematic for NM_000295.5 and NM_000295.4. Transcription start-site of NM_000295.5 is 214 nucleotides downstream of that for NM_000295.4, yielding distinct 5'-UTRs (purple and orange, respectively) (C) Relative abundance of the NM_000295.4 transcript across 6 tissues with the highest overall expression of *SERPINA1*. Abundances of the NM_000295.4 transcript are indicated as a percentage and were obtained from the (50-nt) green (chr14: 94,388,665-94,388,715), red (chr14: 94,388,509-94,388,559), and blue (chr14: 94,383,191-94,383,241) regions, which are unique to the 5'-UTR of NM_000295.4, the 5' intron of the last 5'-UTR exon, and the first CDS exon, respectively. Transcript fractions calculated as a percentage of reads, arithmetic mean (± standard deviation), in the green and red regions compared to the first CDS exon (blue) (D) Violin plot of read depths in the green and red regions normalized to first CDS exon. Significance (p-values) calculated from one-tailed Wilcoxon Rank Sum test.

Figure S2. Structures of 5'-UTR and initial CDS for the endogenous NM_000295.4 transcript and for NM_000295.4 expressed from plasmid-based reporter under cell-free and in-cell conditions. SHAPE reactivity profiles (top) and secondary structure models (bottom) for the NM_000295.4 5'-UTR and 240 nucleotides of the CDS for the endogenous transcript expressed in HepG2 cells and for transcripts expressed from a reporter plasmid in HEK293T cells, under cell-free and in-cell conditions. For the in-cell condition, positions showing high inter-replicate variability (>50%) in the endogenous RNA are shown as short grey bars. Arcs connect modeled base pairs and are colored by pairing probability. The genomic position of the transcription start site for NM_000295.4 is +1.

Figure S3. Principal component analysis of RNA structure groups based on pairing probability. Principal component analysis of similarity in pairing probability for the native sequence transcript and all mutant transcripts (left panel) and inertia versus *k* values, based on *k*-means clustering analysis (right panel) for (A) replicate 1 and (B) replicate 2. Centroids of each group identified by *k*-means clustering are indicated with red x symbols. Native sequence

is marked by a red circle; mutants are identified by number. Groupings were reproducible across replicates.

Figure S4. Energetic costs of unfolding RNA structures at translation initiation site, as a function of window size. The non-equilibrium unfolding model ($\Delta G^{\ddagger}_{unfold}$) is the cost of disrupting RNA structure over a given window, without allowing RNA refolding. Arithmetic means (±standard deviation) for $\Delta G^{\ddagger}_{unfold}$ for symmetric windows of the indicated size, centered at the adenosine of the start codon. *, $p \leq 0.05$; **, $p \leq 0.01$ (two-tailed t-test). This analysis confirms that differences in $\Delta G^{\ddagger}_{unfold}$ are robust to specific choice of window size.

Figure S5. Illustration of importance of SHAPE data for structural modeling of NM_000295.4 transcript. (A) Pairing probability diagrams for structural models derived with (*top*) and without (*bottom*) SHAPE data for the native sequence and representative mutant transcripts. Sens and ppv were calculated from pairing probabilities (≥0.1 threshold). Mutants are the same as shown in **Figure 4**. (B) Principal component analysis for models calculated without SHAPE data. Non-native mutants are labeled to illustrate shifts into new structural groups, relative to those shown in **Figure S3**. Italicized mutants emphasize native-like mutants now classified into non-native structure groups. (C) Analysis analogous to that performed in **Figure 6**, but without using SHAPE data to model RNA structure.

Figure S6. RNA structure at alternative (non-AUG containing) regions is not correlated with translation. (A) Locations of 30-nucleotide regions in spliced NM_000295.4 transcript for comparison with unfolding of the region centered on the start codon (as shown in **Figure 6**). Midpoint of each region is labeled; 30-nucleotide windows (± 15 nucleotides from midpoint) are drawn to scale. (B-D) Analysis analogous to that performed in **Figure 6** shows no correlation between relative translation and $\Delta G^{\ddagger}_{unfold}$ for regions that do not include the AUG start site.

Figure S1

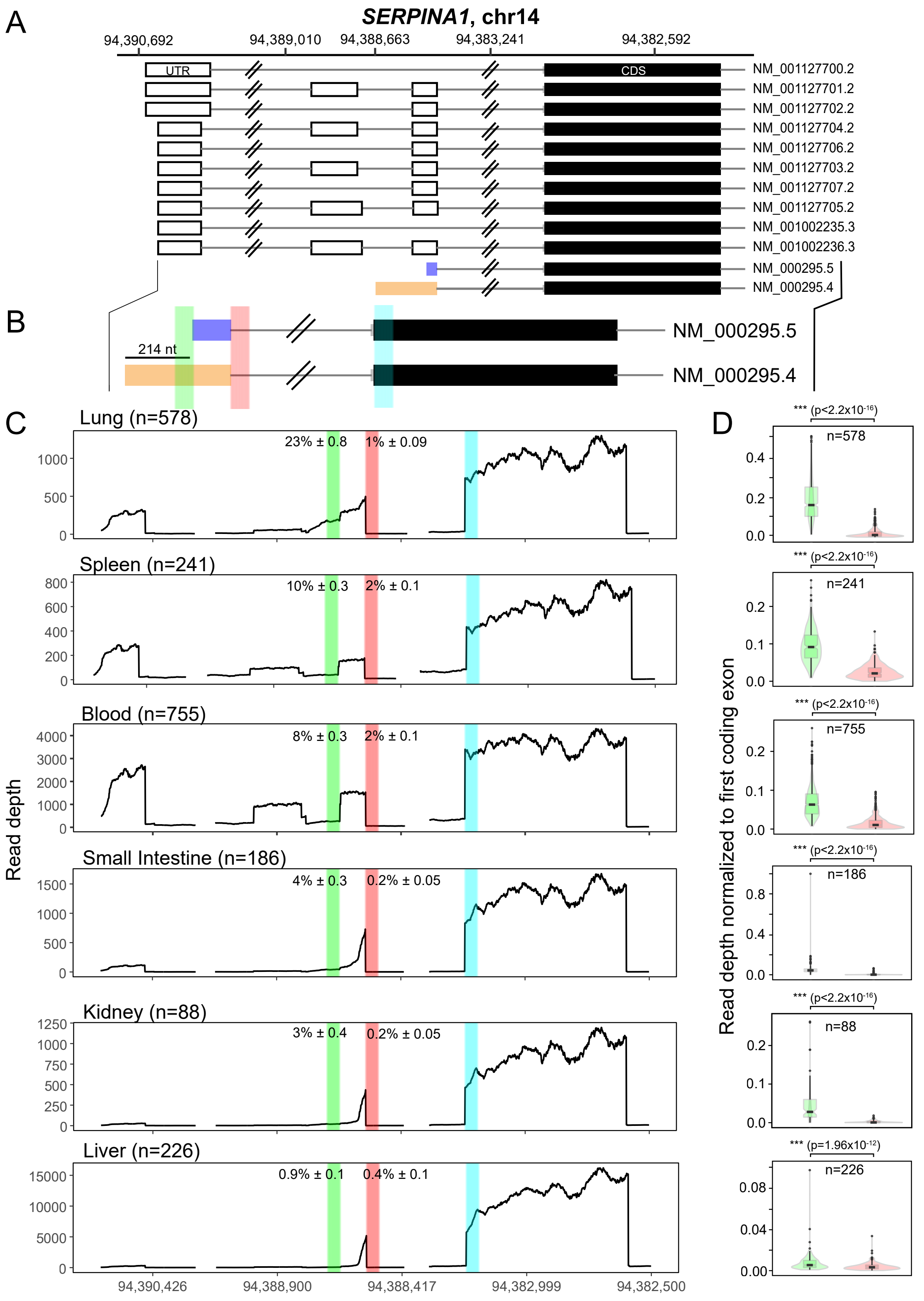

A
SERPINA1, chr14
94,390,692
94,389,010
94,388,663
94,383,241
94,382,592
UTR
CDS
NM_001127700.2
NM_001127701.2
NM_001127702.2
NM_001127704.2
NM_001127706.2
NM_001127703.2
NM_001127707.2
NM_001127705.2
NM_001002235.3
NM_001002236.3
NM_000295.5
NM_000295.4
B
214 nt
NM_000295.5
NM_000295.4
C
Read depth
Lung (n=578)
23% ± 0.8
1% ± 0.09
Spleen (n=241)
10% ± 0.3
2% ± 0.1
Blood (n=755)
8% ± 0.3
2% ± 0.1
Small Intestine (n=186)
4% ± 0.3
0.2% ± 0.05
Kidney (n=88)
3% ± 0.4
0.2% ± 0.05
Liver (n=226)
0.9% ± 0.1
0.4% ± 0.1
94,390,426
94,388,900
94,388,417
94,382,999
94,382,500
D
Read depth normalized to first coding exon
*** (p<2.2x10-16)
n=578
n=241
n=755
n=186
n=88
*** (p=1.96x10-12)
n=226

Figure S2

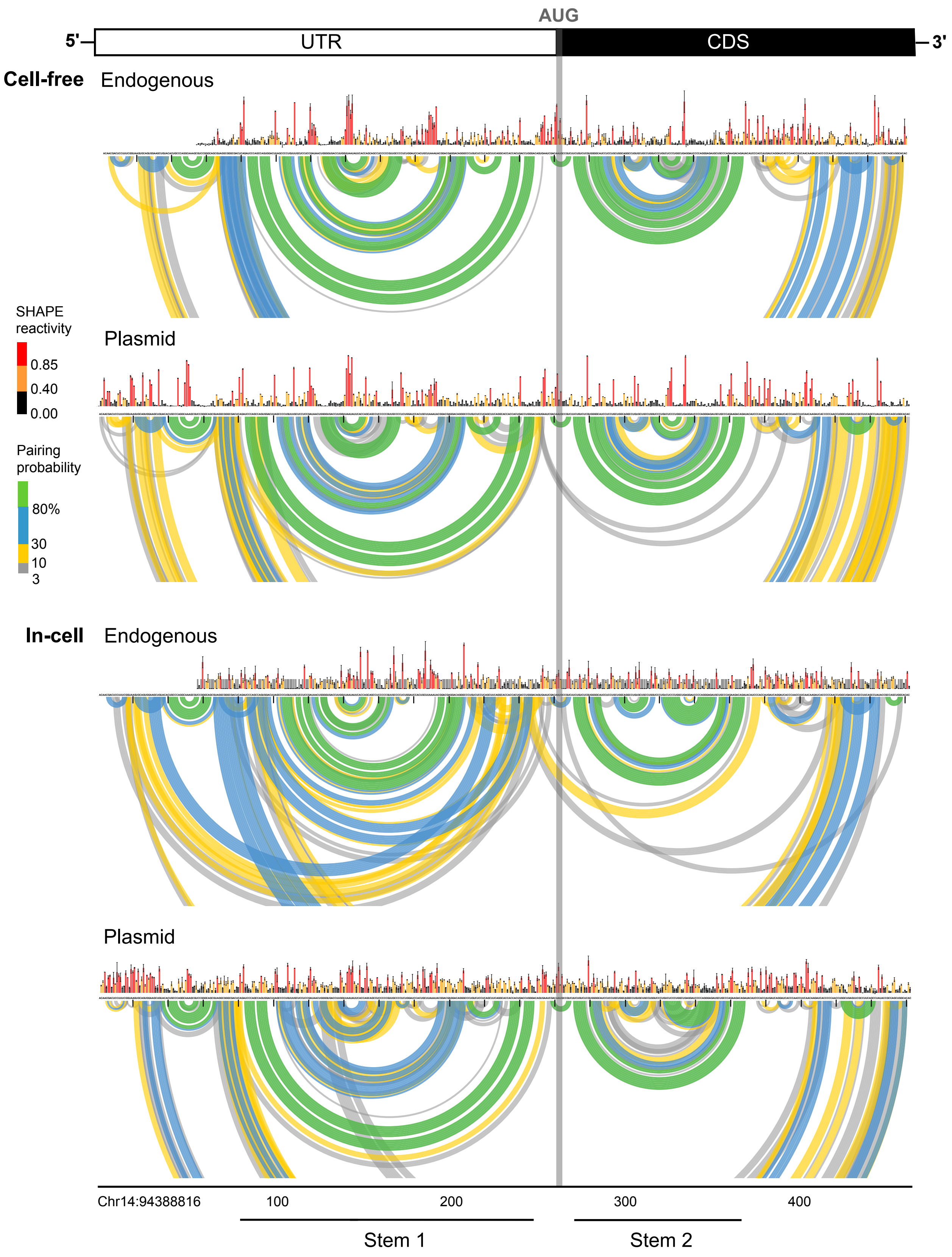

AUG
5'
UTR
CDS
3'
Cell-free
Endogenous
Plasmid
SHAPE reactivity
0.85
0.40
0.00
Pairing probability
80%
30
10
3
In-cell
Endogenous
Plasmid
Chr14:94388816
100
200
300
400
Stem 1
Stem 2

Figure S3

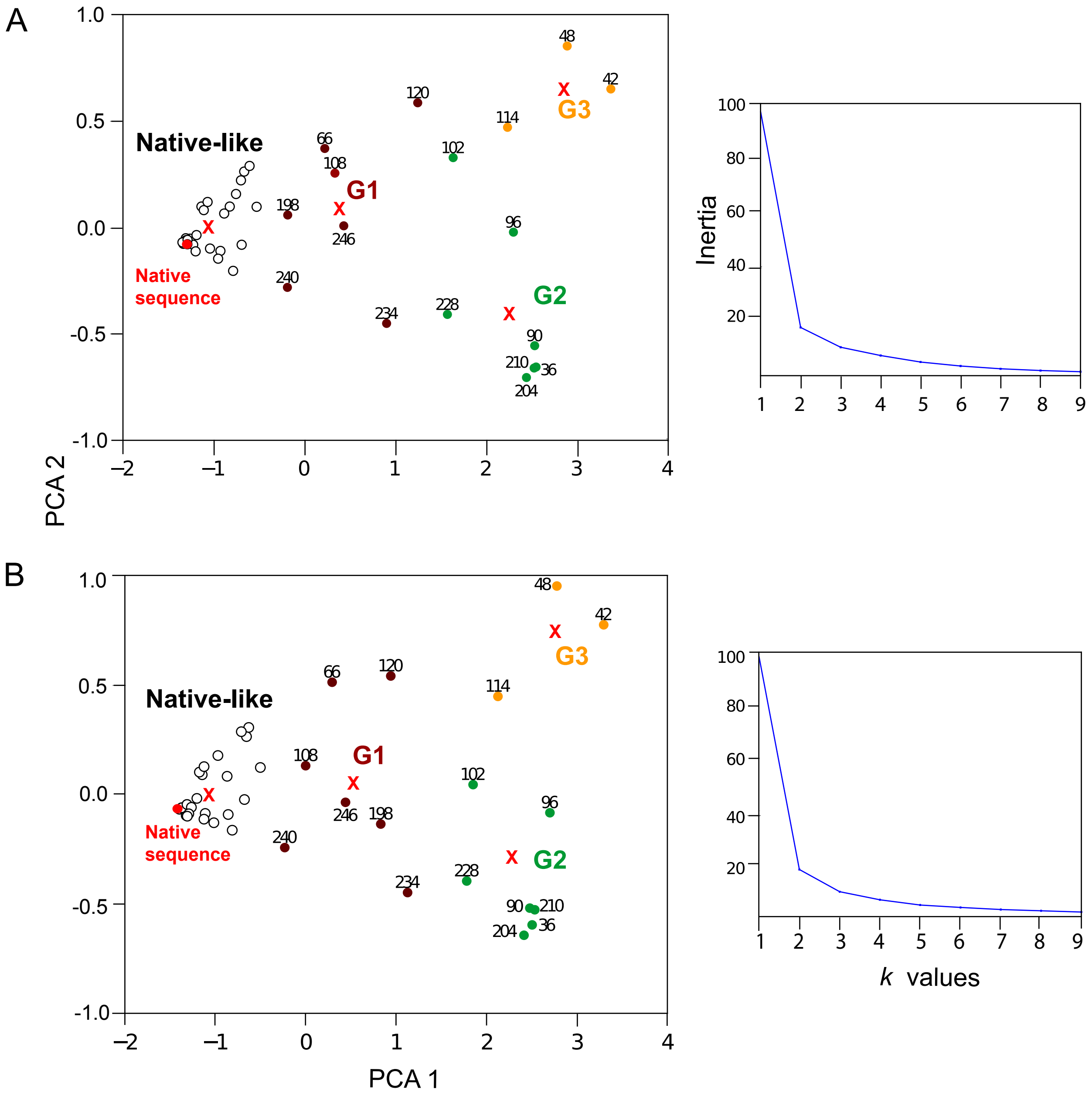
A
Native-like
Native sequence
G1
G2
G3
48
42
120
114
102
66
108
198
246
96
240
234
228
90
210
36
204
PCA 2
Inertia
B
PCA 1
k values

Figure S4

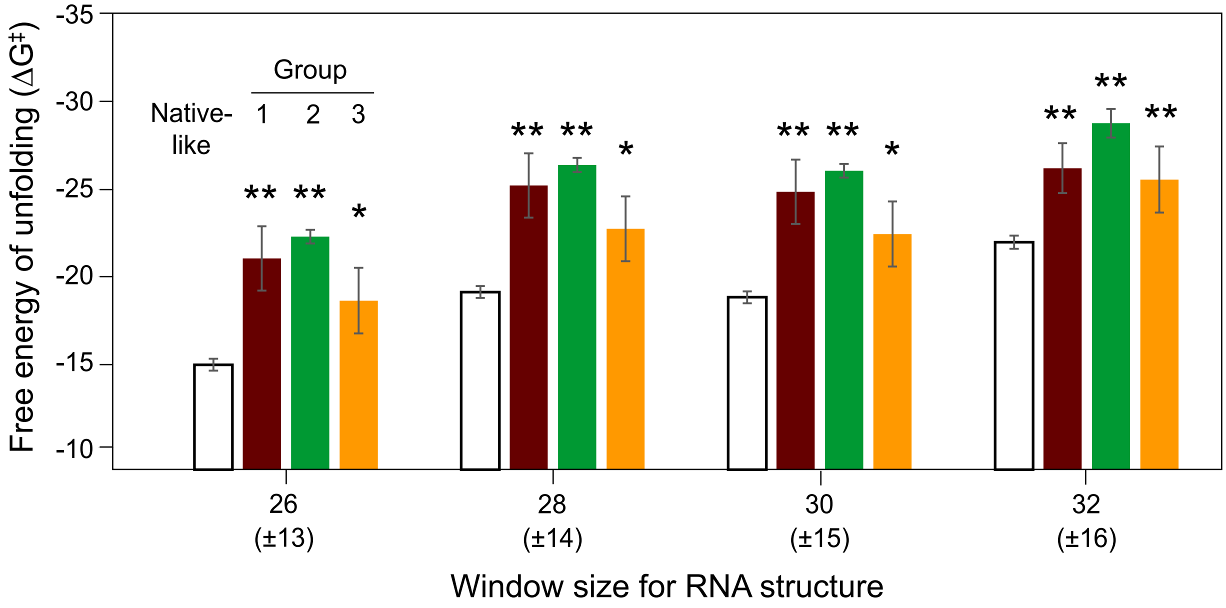

Free energy of unfolding (ΔG‡)
-35
-30
-25
-20
-15
-10
Group
Native-
like
1
2
3
**
**
*
**
**
*
**
**
*
**
**
**
26
(±13)
28
(±14)
30
(±15)
32
(±16)
Window size for RNA structure

Figure S5

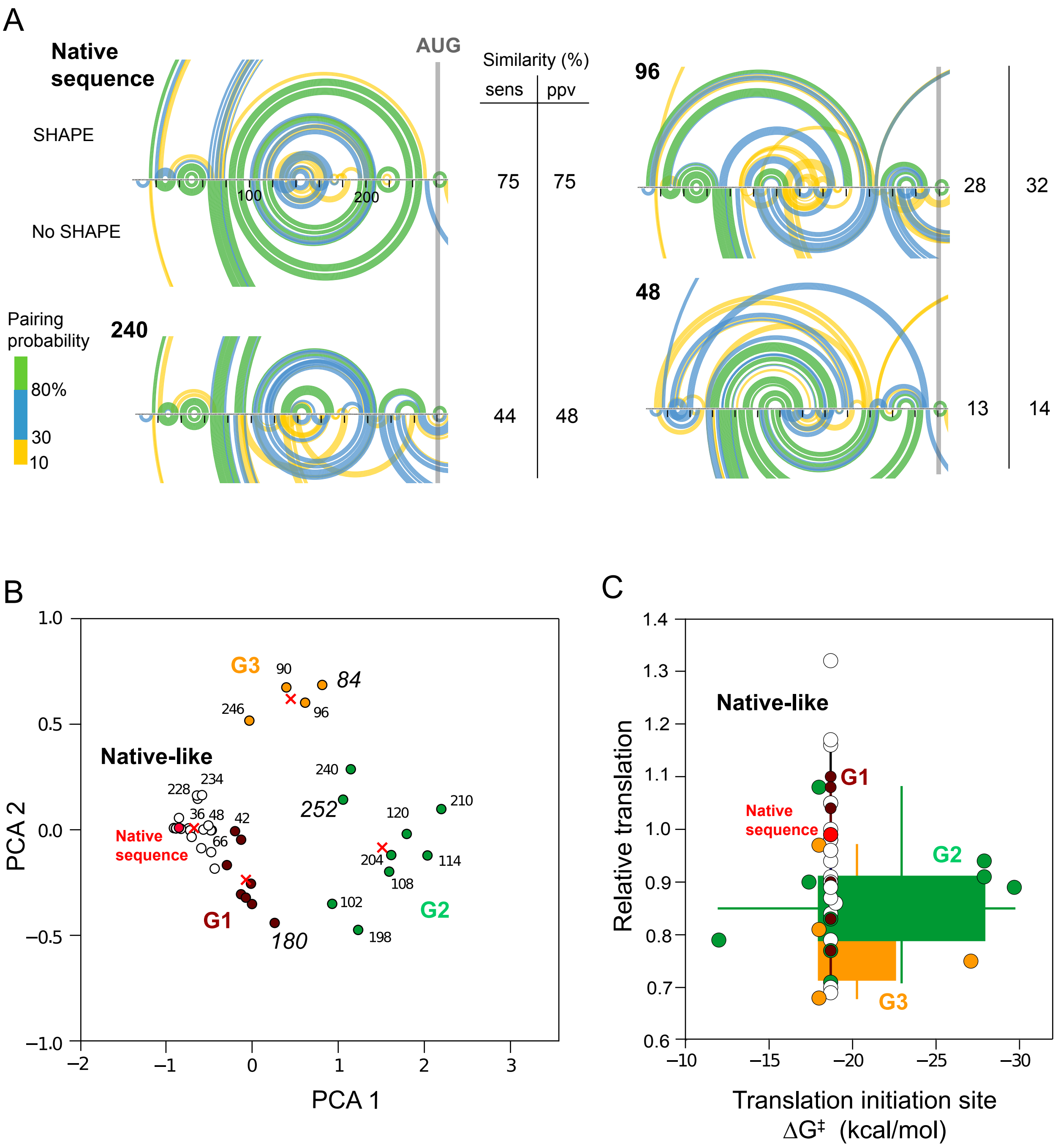

A
Native sequence
AUG
Similarity (%)
sens
ppv
SHAPE
No SHAPE
100
200
75
75
96
28
32
48
13
14
240
44
48
Pairing probability
80%
30
10
B
G3
90
84
96
246
Native-like
240
228
234
252
210
36
48
42
120
Native sequence
66
204
114
108
102
G2
G1
180
198
PCA 1
PCA 2
C
Native-like
G1
Native sequence
G2
G3
Relative translation
Translation initiation site
ΔG‡ (kcal/mol)

Figure S6

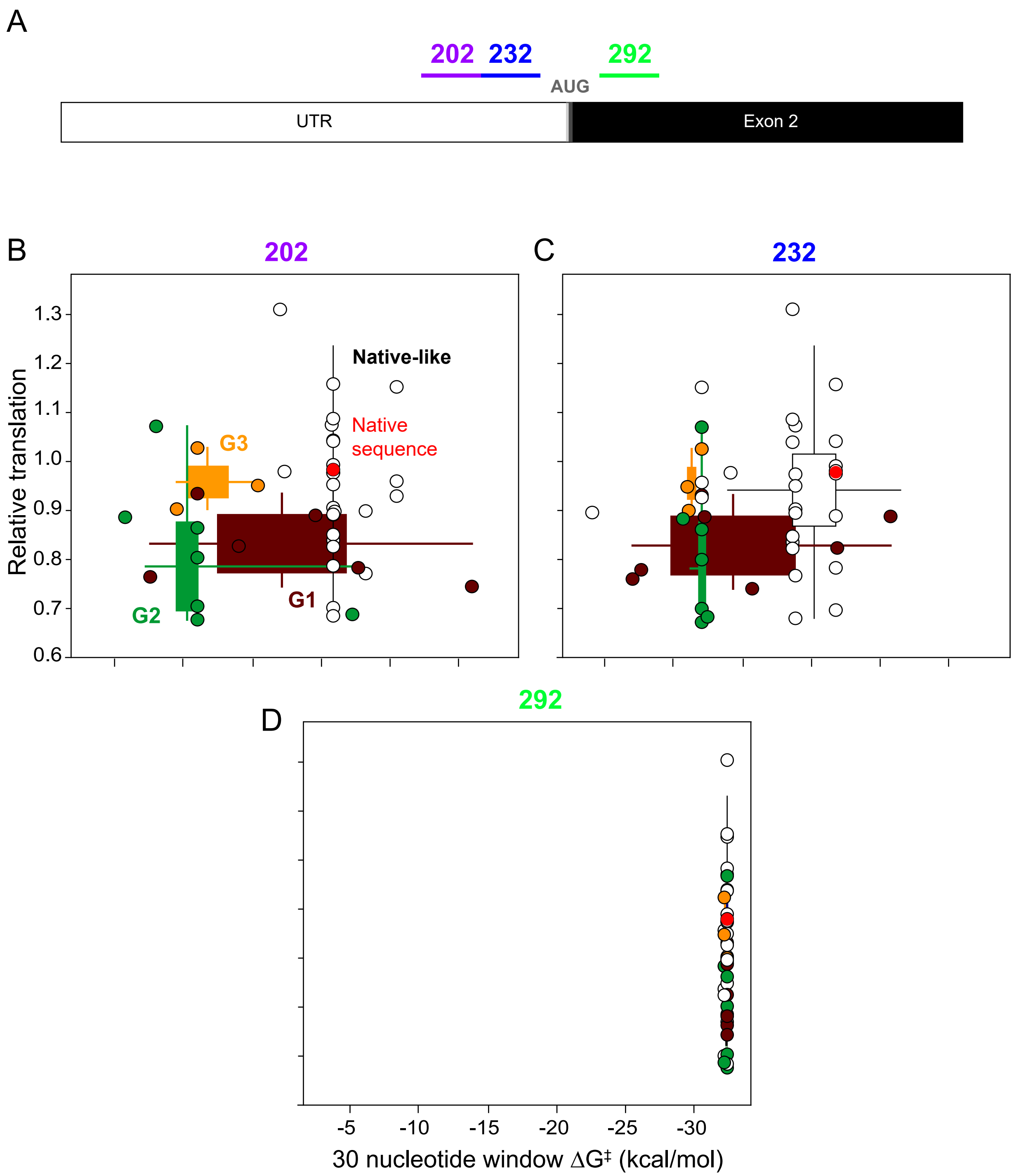

A
202
232
292
AUG
UTR
Exon 2
B
202
C
232
D
292
Relative translation
1.3
1.2
1.1
1.0
0.9
0.8
0.7
0.6
Native-like
Native
sequence
G3
G2
G1
-5
-10
-15
-20
-25
-30
30 nucleotide window ΔG‡ (kcal/mol)

Dataset S1. List of plasmid sequences.

Dataset S2. Spreadsheet containing raw and analyzed translation data from luciferase assays. File also contains modeled thermodynamic stability of structure around the translation start site for all mutants.

Sequencing reads for SHAPE-MaP structural probing data of wild-type and mutant 5'-UTRs are available in the Sequence Read Archive (SRA), Bioproject number **PRJNA749882**.
